# Harnessing the gut microbiome to modulate ferroptosis: a metabolic strategy for the treatment of digestive tract cancers

**DOI:** 10.3389/fimmu.2025.1703964

**Published:** 2025-11-10

**Authors:** Jiexia Wen, Huagang Liang, Min Zhao, Bin Xuan, Xiangcai Meng, Yang Liu, Liwei Wang, Li He, Tao Zhou, Yang Tao, Yimin Wang

**Affiliations:** 1 Department of Central Laboratory, The First Hospital of Qinhuangdao, Qinhuangdao, Hebei, China; 2 Key Laboratory of Research on Molecular Mechanism of Gastrointestinal Tumors in Qinhuangdao, The First Hospital of Qinhuangdao, Qinhuangdao, Hebei, China; 3 State Key Laboratory of Metastable Materials Science and Technology, Yanshan University, Qinhuangdao, China; 4 Department of Thoracic Surgery, The First Hospital of Qinhuangdao, Hebei Medical University, Qinhuangdao, Hebei, China; 5 Department of Pathology, The First Hospital of Qinhuangdao, Hebei Medical University, Qinhuangdao, Hebei, China; 6 Department of General Surgery, The First Hospital of Qinhuangdao, Hebei Medical University, Qinhuangdao, Hebei, China

**Keywords:** ferroptosis, digestive tract tumors, gut microbiota metabolites, tumor microenvironment, cancer therapy

## Abstract

Ferroptosis is a form of regulated cell death defined in recent years, characterized by iron-dependent accumulation of lipid peroxides. A large body of research has demonstrated that ferroptosis is closely associated with the progression of gastrointestinal tumors (such as gastric cancer, colorectal cancer, and esophageal cancer), and gastrointestinal tumor cells exhibit unique sensitivity to ferroptosis. This indicates that ferroptosis has emerged as a highly promising strategy to combat therapy-resistant colorectal cancer. Although the intrinsic ferroptosis-suppressive and ferroptosis-promoting pathways in gastrointestinal tumors have been fully elucidated, the current understanding of the extrinsic metabolites and pathways that regulate ferroptosis in the pathogenesis of gastrointestinal tumors remains relatively limited. Emerging studies have shown a strong link between gut microbial metabolism and the progression of gastrointestinal tumors. This review summarizes the relevant aspects of gut microbiota metabolism, explores how these gut microbiota-derived metabolites regulate cancer progression through ferroptosis, and proposes that targeting gut microbiota-mediated ferroptosis represents a potential therapeutic approach for gastrointestinal tumors.

## 1 Introduction

Ferroptosis, a regulated form of cell death distinct from apoptosis, pyroptosis, and necrosis, has attracted considerable attention since its formal characterization in 2012. It is primarily driven by iron-dependent lipid peroxidation, initiated by the accumulation of peroxidized phospholipids in cellular membranes through the action of intracellular iron (1). Excess iron promotes reactive oxygen species (ROS) generation via the Fenton reaction, overwhelming cellular antioxidant defenses, particularly the glutathione (GSH) and glutathione peroxidase 4 (GPX4) system. When ROS production surpasses the cell’s detoxification capacity, they oxidize unsaturated fatty acids within membrane phospholipids (2), leading to lipid peroxide formation that disrupts membrane integrity and causes extensive cellular damage. Morphologically, ferroptosis is marked by mitochondrial abnormalities such as shrinkage, increased membrane density, and loss of cristae (3). Unlike apoptosis, it does not involve cytoplasmic vesicles, chromatin condensation, or nuclear fragmentation; it also lacks the swelling and organelle disintegration typical of necrosis.

Several small molecules modulate ferroptosis, a form of cell death driven by iron-dependent lipid peroxidation. Compounds such as erastin, sulfasalazine, sorafenib, artemisinin derivatives, and buthionine sulfoximine promote ferroptosis by depleting GSH (4). Others, including RSL3, altretamine, DPI17, and FIN56, directly inhibit GPX4 (5–8). Conversely, ferroptosis can be suppressed by iron chelators (e.g., deferoxamine) or specific inhibitors like ferrostatin-1, liproxstatin-1, vitamin E, and others (9). Key protein regulators, including GPX4, p53, SLC7A11, ACSL4, NOX, and NRF2, also critically influence ferroptosis (10). Additionally, the gut microbiota and its metabolites play a significant role: probiotics and metabolites such as short-chain fatty acids (SCFAs), bile acids, and tryptophan derivatives can inhibit ferroptosis and support intestinal health (11). In contrast, bacterial lipopolysaccharide (LPS) promotes ferroptosis by increasing iron accumulation and oxidative stress, exacerbating tissue damage.

Ferroptosis has been increasingly implicated in the pathogenesis of numerous diseases beyond the digestive system, including neurological disorders, ischemia/reperfusion injury, acute kidney injury, cardiovascular diseases, and various cancers (12). Within the gastrointestinal tract, growing evidence specifically links ferroptosis to esophageal, gastric, pancreatic, and colorectal cancers, as well as intestinal I/R injury and inflammatory bowel disease (13). These findings highlight ferroptosis not only as a key disease mechanism but also as a promising therapeutic target. This review summarizes recent advances in understanding the role of ferroptosis in gastrointestinal diseases, with a special focus on the regulatory influence of the gut microbiota and its metabolites. By exploring how microbial components modulate ferroptosis, we further discuss the potential of targeting ferroptosis as a novel treatment strategy for gastrointestinal disorders.

## 2 Ferroptosis

### 2.1 Mechanisms of ferroptosis

#### 2.1.1 Lipid peroxidation

Lipid peroxidation is a key executioner in ferroptosis, primarily targeting esterified polyunsaturated fatty acids (PUFAs) in membrane phospholipids, while free PUFAs are largely unaffected. The enzymes ACSL4 and LPCAT3 play crucial roles in synthesizing esterified PUFAs and incorporating arachidonic acid into phospholipids. Knocking down either enzyme reduces the formation of esterified PUFAs and increases cellular resistance to ferroptosis (14), indicating that PUFA availability and esterification are prerequisites for ferroptosis initiation (15). Lipoxygenases (LOXs), particularly ALOX15 (16), are central to enzymatic lipid peroxidation in ferroptosis. ALOX15 catalyzes the oxygenation of arachidonic acid, a process enhanced by the scaffold protein PEBP1 (17). This leads to increased lipid peroxide accumulation, which disrupts membrane integrity and promotes ferroptotic cell death.

#### 2.1.2 Abnormal iron metabolism

Abnormalities in iron metabolism are critical for the initiation of ferroptosis. Elevated intracellular iron levels—often due to uptake of exogenous iron sources such as ferric ammonium citrate, ferric citrate, or ferric chloride hexahydrate—sensitize cells to ferroptosis (18). Excess ferrous iron (Fe^2+^) participates in Fenton reactions, generating highly reactive hydroxyl radicals (19). Under physiological conditions, iron homeostasis is tightly regulated. Cellular iron uptake is primarily mediated by transferrin receptor 1 (TFR1), which facilitates transferrin-bound iron endocytosis. Knockdown of *TFR1* has been shown to inhibit ferroptosis induced by erastin or cystine deprivation. Additionally, intracellular iron levels are modulated by ferritinophagy, an autophagy-like process mediated by nuclear receptor coactivator 4 (NCOA4). NCOA4 targets ferritin for degradation, releasing iron into the labile iron pool (LIP), thereby increasing free iron levels and promoting ferroptosis (20).

#### 2.1.3 GSH depletion

GSH is a key metabolic regulator and antioxidant that neutralizes ROS, protecting lipids, proteins, and DNA from oxidative damage and limiting lipid peroxide accumulation. Ferroptosis can be induced by compounds like erastin and RSL3, which impair the GSH-dependent antioxidant system. Erastin inhibits system Xc^−^, a cystine/glutamate antiporter, thereby reducing cystine uptake and subsequent GSH synthesis. RSL3 directly inhibits GPX4, which relies on GSH to eliminate lipid peroxides (21). Together, these effects lead to unchecked lipid peroxidation and ferroptosis. Cystine, the oxidized form of cysteine, is essential for GSH synthesis. It is imported into cells via SLC7A11, a key component of system Xc^−^. Inside the cell, cystine is reduced to cysteine and used for GSH production. By enhancing cystine uptake and GSH synthesis, SLC7A11 helps counteract ferroptosis driven by ROS and iron accumulation. This protective mechanism is often upregulated in tumor cells to promote survival under oxidative stress (22). The cystine/GSH/GPX4 axis is now established as a central pathway regulating ferroptosis, with GPX4 playing a critical role in controlling this cell death process (23).

### 2.2 Inhibitory pathways of ferroptosis

#### 2.2.1 SLC7A11-GSH-GPX4 pathway

System Xc^−^ is an essential amino acid antiporter composed of a light chain (SLC7A11) and a heavy chain (SLC3A2). It mediates the exchange of extracellular cystine for intracellular glutamate, facilitating cystine uptake, a critical step for GSH biosynthesis. Upon import, cystine is reduced to cysteine, the rate-limiting precursor for GSH. As a key antioxidant, GSH neutralizes lipid peroxides and protects cells from oxidative damage that drives ferroptosis. This detoxification is primarily executed by GPX4, which uses GSH to reduce lipid hydroperoxides into nontoxic lipid alcohols, thereby maintaining membrane integrity (24). Inhibiting GPX4 leads to GSH depletion, accumulation of lipid peroxides, and ultimately induces ferroptosis. GPX4 activity depends not only on GSH availability but is also regulated by epigenetic and post-translational mechanisms that modulate its expression and function (25).

#### 2.2.2 CoQ10-FSP1 pathway

Coenzyme Q10 (CoQ10), also known as ubiquinone, is a lipid-soluble antioxidant that inhibits lipid peroxidation in cell membranes. In the CoQ10–FSP1 pathway, ferroptosis suppressor protein 1 (FSP1) reduces CoQ10 to ubiquinol (CoQ10H2) using NAD(P)H as a cofactor. Ubiquinol then scavenges lipid peroxyl radicals, thereby suppressing lipid peroxidation and ferroptosis (26, 27). This mechanism functions independently of the canonical SLC7A11–GPX4 axis. Additionally, CoQ10 is synthesized via the mevalonate pathway; its inhibition decreases both CoQ10 and GPX4 levels, increasing cellular susceptibility to ferroptosis (28).

#### 2.2.3 GCH1-BH4 pathway

The guanosine 5’-triphosphate (GTP) cyclohydrolase-1 (GCH1)–tetrahydrobiopterin (BH4) pathway represents a key GPX4-independent mechanism that inhibits ferroptosis. BH4 serves as an essential cofactor in nitric oxide production, neurotransmitter synthesis, and aromatic amino acid metabolism. Depletion of BH4 causes nitric oxide synthase uncoupling and elevated ROS levels. Together with dihydrobiopterin (BH2), BH4 forms a redox cycle that scavenges endogenous oxidative free radicals, thereby suppressing ferroptosis (29). This cycle is maintained by dihydrofolate reductase (DHFR) using NADP^+^/NAD(P)H as a cofactor (29, 30). Notably, supplementation with BH4, but not BH2, confers protection against ferroptosis.

*De novo* synthesis of BH4 depends on three enzymes: GCH1, 6-pyruvoyltetrahydropterin synthase (PTS), and sepiapterin reductase (SPR). Among these, GCH1 is the rate-limiting enzyme and a central regulator of ferroptosis sensitivity (31). Its deficiency or pharmacological inhibition reduces BH4 levels, leading to oxidative radical accumulation and ferroptosis. Conversely, *GCH1* overexpression enhances BH4 biosynthesis, reduces ROS, and promotes CoQ10 production, via synthesis of the precursor 4-hydroxybenzoate, further increasing ferroptosis resistance (30, 32). Thus, the GCH1–BH4 pathway constitutes an important endogenous antioxidant system that inhibits ferroptosis independently of the GPX4-GSH axis.

#### 2.2.4 MBOAT1/2-MUFA pathway

Lipid peroxidation, a key executor of ferroptosis, primarily targets esterified PUFAs within membrane phospholipids. Ferroptosis can be inhibited by monounsaturated fatty acids (MUFAs), which are produced by SCD1 and counteract the detrimental effects of PUFAs (33). Through genome-wide CRISPR activation screens, Liang et al. identified membrane-bound O-acyltransferase 1/2 (MBOAT1/2) as a novel ferroptosis inhibitor. MBOAT1/2 suppresses ferroptosis by remodeling phospholipids via a mechanism independent of GPX4 or FSP1. Specifically, ACSL3 activates MUFAs to form MUFA-CoA, which MBOAT1/2 then uses to synthesize MUFA-containing phospholipids (MUFA-PLs) by transferring the acyl group to lyso-phosphatidylethanolamine (lyso-PE). By replacing oxidizable PUFAs in membranes, MUFA-PLs reduce lipid peroxidation and inhibit ferroptosis (34). The expression of MBOAT2 is regulated by the androgen receptor (AR). AR antagonists lower MBOAT2 levels, and combining them with ferroptosis inducers potently suppresses tumor growth in AR^+^ prostate cancer. Similarly, MBOAT1 is highly expressed in estrogen receptor (ER)-associated cancers, such as breast, ovary, and endometrium, and is likely regulated by ER. ER antagonists reduce MBOAT1 expression and sensitize cells to ferroptosis inducers (35). Thus, sex hormone receptors play a critical role in regulating ferroptosis.

#### 2.2.5 Mitochondrial DHODH pathway

**Figure 1:**
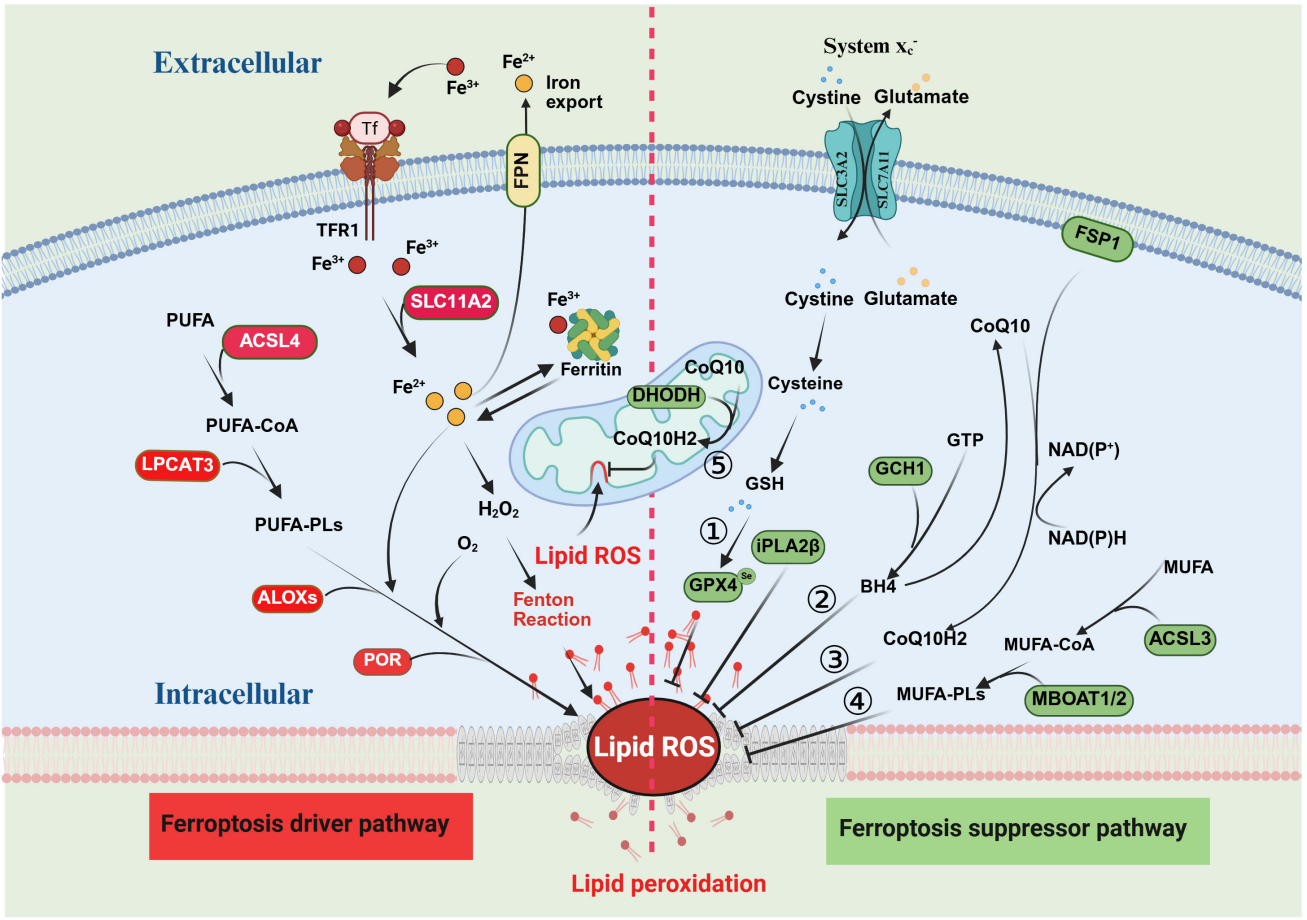
Mechanisms of ferroptosis and suppression. The red section on the left side of the figure illustrates the mechanism of ferroptosis, with proteins in the red boxes representing the key promoters of this process. The green section on the right depicts the major inhibitory pathways of ferroptosis, where proteins in the green boxes denote the main inhibitors. There are five principal inhibitory pathways for ferroptosis: 1) the System Xc^−^–GPX4 pathway; 2) the CoQ10–FSP1 pathway; 3) the GCH1–BH4 pathway; 4) the MUFA pathway; and 5) the mitochondrial DHODH pathway.

Dihydroorotate dehydrogenase (DHODH) is a mitochondrial enzyme located on the outer surface of the inner mitochondrial membrane (IMM) and plays a role in important metabolic pathways such as cytochrome P450, purine synthesis, and fatty acid metabolism (36, 37). Studies have shown that the DHODH pathway can protect cells from oxidative stress and mitochondrial damage, including downregulating ROS levels, maintaining mitochondrial membrane potential, and inhibiting apoptosis, which has been proven to be a novel defense system against mitochondrial ferroptosis (38, 39). DHODH regulates mitochondrial lipid peroxidation and ferroptosis by catalyzing ubiquinone (CoQ10)-mediated oxidation of dihydroorotate (DHO) to orotate (OA) and reducing CoQ10 to ubiquinol (CoQ10H2) (38, 40). DHO protects cells from RSL3-induced ferroptosis, and the DHODH inhibitor brequinar (BRQ) is able to promote ferroptosis in GPX4-deficient cancer cells. RSL3-induced ferroptosis in DHODH-deficient cells is not spared by activation of FSP1, suggesting that DHODH acts in an FSP1-independent manner. In addition, studies have shown that DHODH can work in parallel with mitochondrial GPX4 to inhibit ferroptosis (41, 42). Inhibition of DHODH significantly induced mitochondrial lipid peroxidation and ferroptosis in cancer cells with low GPX4 expression, but only made cancer cells with high GPX4 expression sensitive to ferroptosis. In cells with *GPX4* knockdown, it was confirmed that mitochondrial GPX4 rather than cytoplasmic GPX4 was able to make cells more resistant to ferroptosis. Therefore, these findings suggest that DHODH and GPX4 act as redundant defense mechanisms in mitochondria to prevent ferroptosis (38) (**Figure 1**).

## 3 Intestinal flora and metabolites in the regulation of ferroptosis

Ferroptosis is characterized by iron overload, excessive ROS production, elevated levels of PUFAs, and extensive lipid peroxidation. Growing evidence highlights a strong relationship between the gut microbiota and ferroptosis. Intestinal probiotics may exert protective effects against ferroptosis by chelating metal ions, scavenging or reducing ROS, and modulating key ferroptosis-related enzymes (43, 44). In addition, microbial metabolites such as SCFAs, bile acids, and tryptophan derivatives are increasingly recognized as critical regulators of this process (45). As summarized in **Table 1**, these compounds influence ferroptosis through distinct mechanisms.

**Table 1:** Microbial compositions and metabolites associated with ferroptosis.

Microorganisms and metabolites that suppress ferroptosis		
Microorganisms/Metabolites	Functions related to ferroptosis	References
*Lactobacillus reuteri*	Inhibits HIF-2α expression through the production of reuterin, limiting intestinal iron absorption and balancing iron homeostasis.	(46)
*Bifidobacterium*/*Lactobacillus*	1) Reduces intestinal pH via SCFAs production, improving iron bioavailability and avoiding intestinal iron overload during oral iron supplementation; 2) By expressing FeoB protein and siderophores, inorganic iron is converted into organic iron to promote iron absorption and reduce the damage of free iron to the intestine.	(47–49)
*E.coli*	Removal of Fenton reaction substrates, assimilation of Fe^2+^, and decomposition of H_2_O_2_.	(50, 51)
*Lactobacillus acidophilus*/*Bacillus coagulans*/*Lactococcus lactis*	Activation of the NRF2/HO-1 pathway regulates HO-1 expression (generally protective).	(51, 52)
*Lactobacillus plantarum*	Converts linoleic acid into conjugated linoleic acid. Activates the NRF2-ARE pathway to promote the expression of antioxidant genes.	(53)
*Acetobacter* spp./Yeasts/*Lactobacilli*	Inhibits 15-lipoxygenase (15-LOX) activity.	(54)
*Lactococcus lactis*	Downregulates ACSL4, upregulates FSP1, and inhibits ferroptosis through the Keap1/NRF2/GPX4 pathway.	(55)
Butyrate	1) Activates the P21/NRF2/NF-κB pathway, inhibits NOX2 expression, and upregulates SOD activity; 2) Improves mitochondrial function via the PGC-1α signaling pathway, enhancing oxidative phosphorylation and β-oxidation; 3) Activates NRF2 through the AMPK–ULK1–p62 signaling pathway, alleviating ferroptosis in acute liver injury.	(56–60)
Acetate	Reduces NOX2 expression in T cells and inhibits HDAC activity to prevent ROS accumulation.	(61)
Tryptophan Metabolites	1) 5-HT and 3-HA are effective RTAs, reducing radical accumulation; 2) IDA activates the AHR-ALDH1A3 axis, promoting FSP1-mediated generation of reduced CoQ10; 3) Activate the NRF2 signaling pathway.	(50, 62–65)
Urolithin A	Enhances mitophagy to clear dysfunctional mitochondria (a major source of ROS) and reduces excessive inflammation.	(66)
Bile Acids	1) Activates FXR, inhibiting lipid peroxidation and ferroptosis by upregulating the expression of ferroptosis-related genes such as GPX4, FSP1, SCD1, and ACSL3. 2) Activates VDR, inhibiting ferroptosis through pathways like NRF2/GPX4 and NRF2/HO-1	(67–70)
Vitamin K (Reduced form VKH_2_)	Inhibits lipid peroxidation in an FSP1-dependent manner.	(71)
L-lactate	Inactivates AMPK, subsequently upregulates SREBP1/SCD1, and promotes the generation of anti-ferroptotic MUFAs.	(65)
Folate (VB9)	Inhibits ferroptosis primarily by reducing glutamate levels.	(72)
Cobalamin (VB12)	Inhibits ferroptosis by inhibiting the SBP-1/SREBP1 lipogenesis axis and reducing lipid peroxidation.	(73)
Vitamin D (VD)	Activates VDR to regulate the NRF2–GPX4 and NRF2–HO-1 signaling pathways, thereby inhibiting ferroptosis.	(69, 74)
Vitamin E (as α-tocopherol)	Directly scavenges lipid peroxides, independent of the GPX4-GSH system.	(75)

Microorganisms and metabolites that promote ferroptosis		
Microorganisms/Metabolites	Functions related to ferroptosis	References
*Mycobacterium tuberculosis*	Elevated HO-1 levels increase intracellular iron and ROS production, exceeding the cell’s antioxidant compensatory capacity.	(76)
*Pseudomonas aeruginosa*	1) Alters the host’s overall lipid metabolic profile by affecting fatty acyl-CoA ligases; 2) Utilizes host 15-LOX to directly cause ferroptosis.	(77, 78)
*Bacillus Calmette-Guérin (BCG)*	Promotes ferroptosis by downregulating GPX4 and FSP1.	(79)
Lipopolysaccharide (LPS)	1) Triggers inflammatory response and oxidative stress by activating TLR4, leading to a significant increase in intracellular ROS levels. 2) Upregulates ACSL4 expression via specificity protein 1 (Sp1).	(80, 81)
Deoxycholic Acid (DCA)	Significantly promotes iron absorption and accumulation in intestinal cells by upregulating HIF-2α and its downstream DMT1, thereby increasing cellular susceptibility to ferroptosis.	(82) (83)
Glycodeoxycholic Acid (GDCA)	Activates TFR-ACSL4-mediated ferroptosis, increases fecal lipid content, and reduces intestinal lipid digestibility.	(84)
Propionate	Promotes ACSL4-regulated ferroptosis.	(70)
Butyrate	1) Downregulates GPX4 and SLC7A11 via the FFAR2–mTORC1 axis; 2) Enhances erastin-induced ferroptosis by regulating the ATF3–SLC7A11 pathway in certain cancer cells.	(85–88)
D-lactate	Promotes ferroptosis by downregulating the anti-ferroptosis gene SLC7A11 and reducing GSH synthesis.	(89)
Vitamin B2 (Riboflavin)	Under specific conditions (e.g., forming nanocomplexes with iron ions), it can synergistically promote iron accumulation and ROS generation.	(90)
Vitamin D (VD)	Induce ferroptosis by upregulating ATF3 transcription to reduce SLC7A11 expression.	(91)

### 3.1 Regulation of iron accumulation by intestinal flora and metabolites

Excess free iron, owing to its redox activity, drives ROS generation through the Fenton reaction and serves as a core trigger of ferroptosis. The intestinal microbiota and their metabolites in the regulation of host iron homeostasis via intricate and multilayered networks that complement classical iron metabolic pathways. Certain microbial metabolites directly target key nodes of iron metabolism. For instance, deoxycholic acid (DCA), a secondary bile acid produced by *Bacteroidetes* such as *Clostridium scindens (*82), significantly enhances iron absorption and accumulation in intestinal epithelial cells by upregulating hypoxia-inducible factor-2α (HIF-2α) and its downstream effector, divalent metal transporter 1 (DMT1). This mechanism is independent of classical antioxidant systems such as the GPX4-GSH axis but increases cellular susceptibility to ferroptosis by elevating the “iron substrate” necessary for its initiation (83). Conversely, *L. reuteri* balance this by inhibiting HIF-2α expression to limit intestinal iron absorption through the production of reuterin, demonstrating the fine-tuned balance of iron homeostasis within the microbial community (46).

On the other hand, the gut microbiota and their metabolites can indirectly modulate ferroptosis by regulating systemic iron distribution. They influence plasma ferritin levels (80) and promote the expression of iron-handling molecules such as ferredoxin and hepcidin (92). Together with macrophage-mediated iron recycling, these effects contribute to maintaining iron homeostasis and the stability of systemic iron cycling. In patients with anemia, oral iron supplementation (e.g., FeSO_4_) has been shown to induce ferroptosis-related intestinal injury (93). Co-administration of probiotics such as *Bifidobacterium* and *Lactobacillus* can mitigate this effect by producing SCFAs, which lower intestinal pH and enhance iron bioavailability. This not only corrects anemia more efficiently but also prevents intestinal iron overload and ferroptosis (47). Such probiotic-mediated protection may serve as a compensatory mechanism that alleviates the oxidative stress imposed by iron supplementation on the classical system Xc^−^–GSH–GPX4 antioxidant axis, primarily by optimizing the local intestinal microenvironment. Furthermore, commensal bacteria can fine-tune iron absorption and utilization by expressing FeoB proteins and siderophores, or by converting inorganic iron into more bioavailable organic forms (48, 49).

Importantly, under conditions of iron overload, the gut microbiota can also confer cellular protection independent of iron metabolism by generating metabolites with intrinsic antioxidant activity. A notable example is the tryptophan-derived metabolite 3-hydroxyanthranilic acid (3-HA), a potent radical-trapping antioxidant. Functionally analogous to FSP1, 3-HA directly scavenges lipid radicals in a manner independent of the GPX4 and CoQ10 systems. Therefore, even when metabolites such as DCA promote intracellular iron accumulation, 3-HA can directly inhibit the execution phase of lipid peroxidation through this parallel antioxidant pathway, thereby preventing the onset of ferroptosis (50, 62). Collectively, these findings illustrate a “multi-level, multi-target” regulatory strategy employed by the gut microbiota to modulate ferroptosis, acting both upstream by controlling iron availability and downstream by directly suppressing lipid peroxidation.

**Figure 2:**
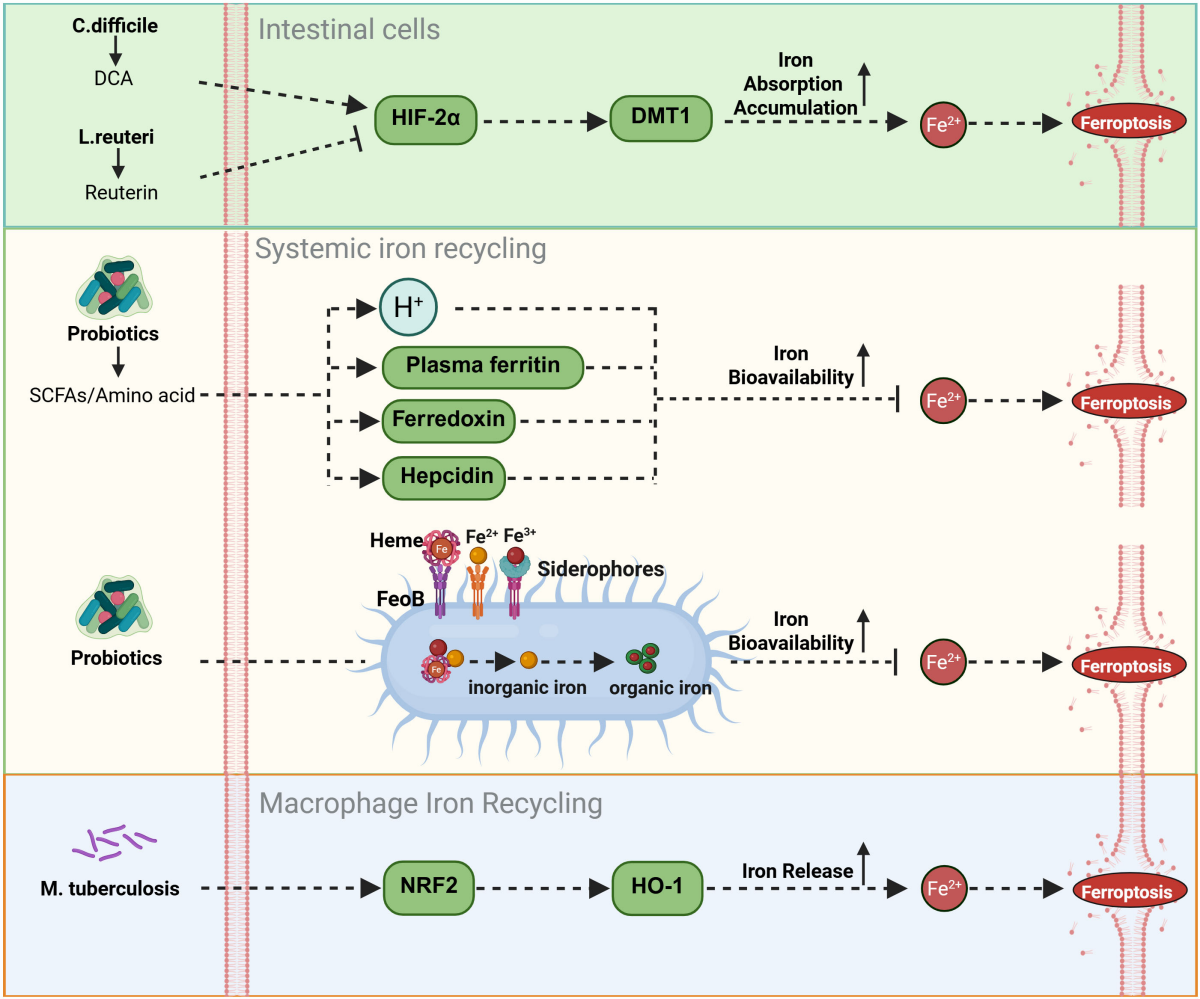
Modulation of iron deposition by gut flora and metabolites. The gut microbiota orchestrates iron metabolism through coordinated mechanisms: at the enterocyte level, Bacteroidetes-derived DCA activates the HIF-2α/DMT1 axis for iron absorption, counterbalanced by L. reuteri-derived reuterin; systemically, probiotics enhance bioavailability via SCFAs/amino acids-induced pH reduction and modulate ferritin/ferredoxin/hepcidin for homeostasis; further precision is achieved via FeoB/siderophore-mediated iron conversion, while macrophage iron recycling is activated through the NRF2/HO-1 pathway.

During macrophage-mediated iron recycling, activation of the NRF2/HO-1 pathway activated by probiotics generally exerts protective effects, for example, alleviating renal ischemia-reperfusion injury (51) and mitigating pathology in neurodegenerative disease models (52). The induction of HO-1 represents a key adaptive metabolic node, where bilirubin, a product of HO-1 activity, acts as an endogenous antioxidant that can partially compensate for diminished GSH function. However, this pathway exhibits dual roles. Under specific pathological conditions, such as *Mycobacterium tuberculosis* (MTB) infection, excessive HO-1-mediated iron release may overwhelm the cell’s antioxidant defenses, leading instead to increased lipid peroxidation and ferroptosis (76) (**Figure 2**).

### 3.2 Regulation of intestinal flora and metabolites on ROS accumulation

**Figure 3:**
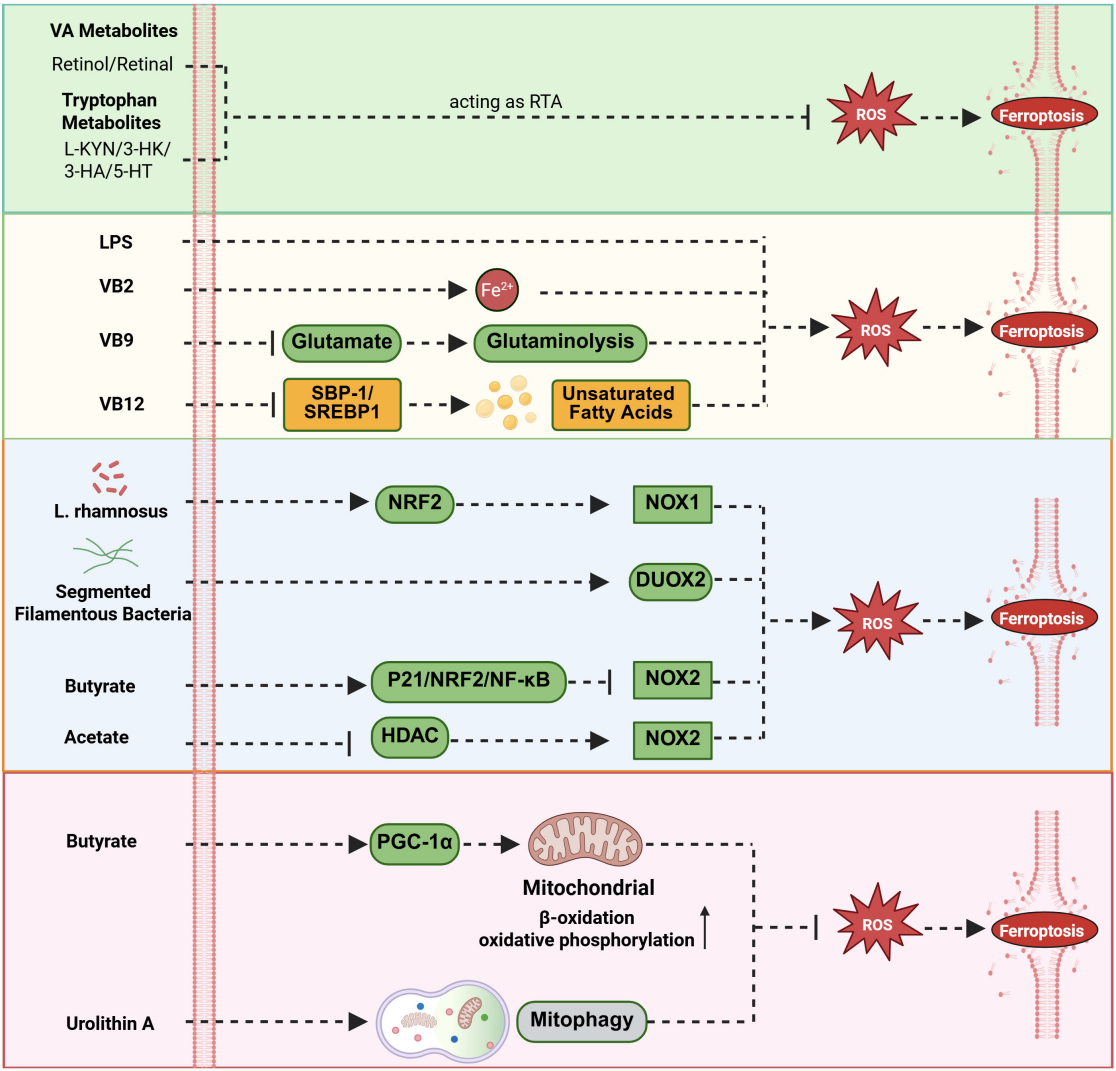
Modulation of ROS accumulation by gut flora and metabolites. The gut microbiota and metabolites regulate intracellular ROS homeostasis and influence the core mechanisms of ferroptosis through a multi-level, interconnected complex network. Vitamin A and tryptophan metabolites act as radical-trapping antioxidants (RTA) to inhibit ferroptosis; LPS significantly increases intracellular ROS levels by activating Toll-like receptor 4 (TLR4) on immune cells such as macrophages; Riboflavin (VB2) promotes iron accumulation and ROS generation, thereby enhancing ferroptosis; Folate (VB9) blocks ferroptosis by reducing glutamate levels; Cobalamin (VB12) inhibits ferroptosis by suppressing the SBP-1/SREBP1 lipogenesis axis; L. rhamnosus upregulates NOX1 expression via the NRF2 pathway; Segmented filamentous bacteria enhance DUOX2 expression levels; conversely, butyrate inhibits NOX2 expression by activating the P21/NRF2/NF-κB pathway, and acetic acid downregulates NOX2 through HDAC inhibition; Regarding the regulation of mitochondrial function, butyrate activates the PGC-1α pathway to enhance mitochondrial function, while urolithin A promotes mitophagy to clear dysfunctional mitochondria and reduce abnormal ROS production.

The intestinal flora and their metabolites participate contribute to maintaining intracellular redox homeostasis through complex regulatory networks, thereby playing a critical role in ferroptosis (94). These regulatory mechanisms are multifaceted, involving direct ROS scavenging, modulation of endogenous antioxidant pathways, or regulation of ROS generation sources (**Figure 3**).

#### 3.2.1 Metabolites that directly scavenge ROS

A variety of microbial metabolites can function as radical-trapping antioxidants (RTAs), directly neutralizing lipid radicals and thereby inhibiting lipid peroxidation. The actions of these metabolites operate in parallel with classical enzymatic systems such as GPX4 and FSP1, forming a rapid, front-line defense against oxidative damage. Vitamin A derivatives (retinol and all-trans-retinal) and several products of the tryptophan metabolic pathway, including l-kynurenine (L-KYN), 3-hydroxykynurenine (3-HK), 3-HA, and serotonin (5-HT), have all been identified as effective RTAs (50, 75, 95, 96). Functionally analogous to FSP1, these molecules directly quench free radicals within the lipid peroxidation chain reaction, independently of the GPX4–GSH axis, thereby constituting an autonomous and parallel layer of protection against ferroptosis.

#### 3.2.2 Metabolites influencing ROS accumulation

Various microbial metabolites directly or indirectly regulate intracellular ROS levels, thereby profoundly influencing the process of ferroptosis. Among them, LPS, a key component of the cell wall of Gram-negative bacteria, typically triggers a strong inflammatory response and oxidative stress by activating Toll-like receptor 4 (TLR4) on immune cells such as macrophages, leading to a significant increase in intracellular ROS levels. This sharp rise in ROS is an important mechanism for initiating antimicrobial defense in the body, but its excessive accumulation can also cause severe cellular damage (81). Among the vitamin B (VB) metabolites, different members exhibit significant differences in regulating ferroptosis, forming a precise regulatory network. For example, riboflavin (VB2) shows potential value in cancer treatment. Studies have shown that a complex formed by VB2 and ferric chloride encapsulated in nanomaterials can target cancer cells at the tumor site, synergistically promoting the accumulation of iron ions and the generation of ROS within the cancer cells, thereby inducing ferroptosis and effectively inhibiting the growth of breast tumors (90). In contrast, folate (VB9) and cobalamin (VB12) inhibit ferroptosis. Their mechanisms are as follows: folic acid primarily blocks the ferroptosis process by reducing glutamate levels, while cobalamin exerts a protective effect by inhibiting the sterol regulatory element-binding protein (SREBP) binding protein-1 (SBP-1)/SREBP1 lipogenesis axis and reducing lipid peroxidation (72, 73).

#### 3.2.3 Regulation of ROS through NOX enzymes and mitochondrial function

Beyond direct modulation of antioxidant defense systems, the gut microbiota and their metabolites influence ferroptosis susceptibility by regulating the primary sources of ROS generation. With respect to NOX enzyme regulation, *Lactobacillus rhamnosus* has been shown to upregulate NOX1 expression via the NRF2 signaling pathway (97), while segmented filamentous bacteria enhance Dual Oxidase 2 (DUOX2) expression levels (98); Conversely, certain microbial metabolites, particularly SCFAs, exert inhibitory effects on NOX activity. For instance, butyrate suppresses NOX2 expression and increases superoxide dismutase (SOD) activity through activation of the P21/NRF2/NF-κB pathway (56), whereas acetate downregulates NOX2 in T cells via histone deacetylase (HDAC) inhibition (61). In terms of mitochondrial regulation, butyrate enhances oxidative phosphorylation and β-oxidation and improves mitochondrial efficiency through activation of the PGC-1α signaling pathway, collectively reducing ROS production and inhibiting ferroptosis (57–59). Similarly, urolithin A, a metabolite derived from gut microbial metabolism of ellagic acid, mitigates ferroptosis by promoting mitophagy, the selective clearance of dysfunctional mitochondria, which are major ROS generators (66). This represents a compensatory mechanism that indirectly supports classical antioxidant systems by preserving mitochondrial integrity and maintaining organelle homeostasis.

#### 3.2.4 Metabolites that indirectly regulate ROS through antioxidant pathways

A major group of microbial metabolites contributes to ROS homeostasis indirectly by activating intracellular antioxidant signaling pathways or serving as critical metabolic cofactors. Their principal function is to enhance or regulate the classical ferroptosis defense systems. VB2 strengthens the system Xc^−^–GPX4 axis through a mechanism independent of IL-17A (99). Similarly, bile acids such as cholic acid and chenodeoxycholic acid (CDCA) activate the Farnesoid X Receptor (FXR), which upregulates the expression of GSH synthase and GPX4, thereby reinforcing this core antioxidant defense network at the transcriptional level (100, 101). Regarding the FSP1-CoQ10 system, the reduced form of vitamin K (VKH2) acts in an FSP1-dependent manner, identifying VKH2 as a key cofactor in this non-canonical antioxidant pathway (71). In addition, the tryptophan-derived metabolite trans-3-indoleacrylic acid (IDA) activates the aryl hydrocarbon receptor (AHR), induces ALDH1A3 expression, and subsequently promotes FSP1-mediated regeneration of reduced CoQ10, thereby strengthening the FSP1–CoQ10 axis (63). Indole-3-acetic acid (IAA), another AHR agonist, enhances antioxidant capacity via two complementary routes. Notably, it promotes SLC7A11-dependent GSH synthesis (64) and provides NADH necessary for CoQ10 biosynthesis (63), effectively bridging the system Xc^−^–GSH pathway and the CoQ10 regeneration system. Likewise, L-lactate indirectly suppresses ferroptosis by inactivating AMPK, which in turn upregulates SREBP1/SCD1 signaling and promotes the synthesis of anti-ferroptotic MUFAs (65). Through this mechanism, L-lactate supports membrane remodeling to resist lipid peroxidation, complementing the classical ferroptosis defense systems.

In summary, the intestinal microbiota and their metabolites form a multilayered regulatory network that maintains ROS balance. They provide direct antioxidant capacity in parallel with GPX4 and FSP1, modulate classical defense systems via transcriptional and signaling pathways, and control ROS production at its sources through regulation of NOX enzymes and mitochondrial function. Disruption of this intricate network is a critical determinant in the onset of ferroptosis and represents a promising therapeutic target for related diseases.

### 3.3 Regulation of intestinal flora and metabolites on fatty acid metabolism and lipid peroxidation

The gut microbiota and their metabolites play a pivotal role in lipid homeostasis and ferroptosis by modulating fatty acid metabolism, influencing redox balance, and regulating cell death pathways.

#### 3.3.1 Regulation of fatty acid absorption and metabolism

Gut microbes finally regulate host fatty acid metabolism through the biotransformation of dietary fatty acids and the production of bioactive signaling molecules. Certain bacteria, such as *Lactobacillus plantarum*, can convert linoleic acid into conjugated linoleic acid and its active intermediates, thereby activating the host NRF2-ARE antioxidant signaling pathway (53). Similarly, commensal bacteria such as *Lactobacillus intestinalis* metabolize dietary vitamin A into its active derivatives, including retinoic acid (102). These vitamin A metabolites, retinol and all-trans-retinal, not only function as potent RTAs (95) but also generate NADH through the ALDH1A3-catalyzed reaction, providing the reducing power necessary for the FSP1-CoQ10 system (63), In this way, they serve as important metabolic supplements to the canonical FSP1-CoQ10 antioxidant pathway. Other microbial metabolites influence lipid metabolism through distinct mechanisms. Glycodeoxycholic acid (GDCA) promotes ferroptosis by activating the TFR-ACSL4 axis and reducing lipid digestibility (84). LPS upregulates ACSL4 expression via activation of the Sp1 transcription factor in esophageal tissue (80). Additionally, *Pseudomonas aeruginosa* can alter the host’s lipid metabolic profile by modulating the activity of fatty acyl-CoA ligases (77).

#### 3.3.2 Direct regulation of lipid peroxidation

**Figure 4:**
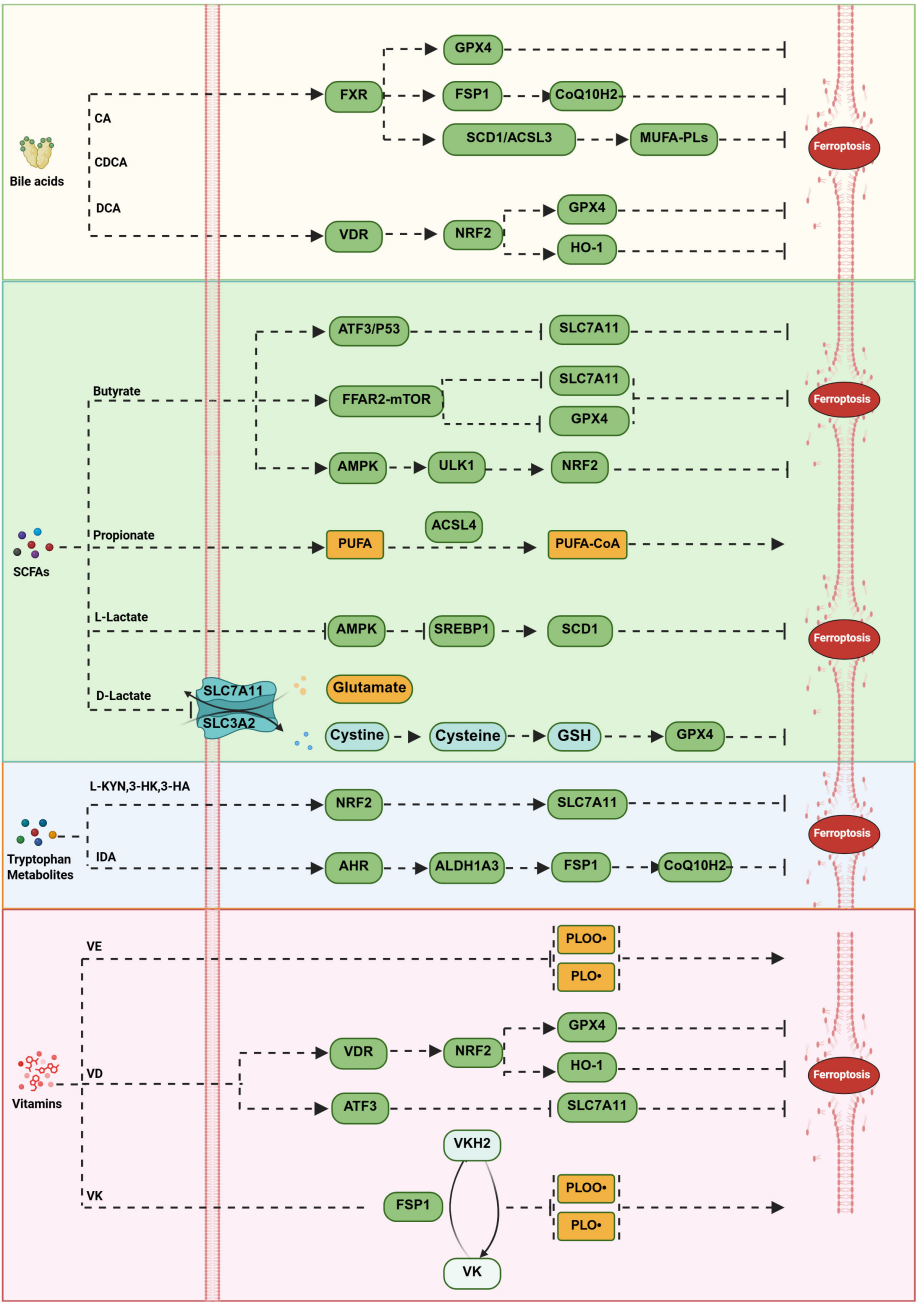
Modulation of lipid peroxidation by gut flora and metabolites. Microbial metabolites regulate lipid peroxidation through multiple mechanisms, forming a complex interaction network with ferroptosis pathways. Bile acids exert dual regulatory effects via nuclear receptors: FXR activation upregulates genes such as GPX4 and FSP1, enhancing the GPX4-GSH and FSP1-CoQ10 systems while inducing SCD1 and ACSL3 to promote the synthesis of monounsaturated fatty acid phospholipids. VDR activation inhibits ferroptosis through pathways like NRF2/GPX4 and NRF2/HO-1. SCFAs exhibit pleiotropic effects: butyrate promotes ferroptosis via the FFAR2–mTORC1 axis by downregulating GPX4 and SLC7A11, mitigates ferroptosis through the AMPK–ULK1–p62 pathway, and also promotes it via ATF3–SLC7A11 and p53–SLC7A11 signaling pathways; propionate promotes ferroptosis by enhancing polyunsaturated fatty acid production and generating more polyunsaturated fatty acid-CoA through ACSL4 action; L-lactate inhibits ferroptosis by suppressing AMPK and upregulating SREBP1 and its downstream target SCD1, whereas D-lactate promotes ferroptosis by downregulating the antiferroptotic gene SLC7A11 and reducing GSH synthesis. Tryptophan metabolites exert protective effects through multiple mechanisms: kynurenine pathway products (L-KYN, 3-HK, and 3-HA) activate NRF2-mediated SLC7A11 expression to prevent ferroptosis; IDA inhibits ferroptosis by enhancing the FSP1-CoQ10 system via the AHR-ALDH1A3 signaling axis. Vitamin metabolites constitute an antioxidant defense system: vitamin E, converted to α-tocopherol, directly scavenges lipid peroxides; VD inhibits ferroptosis through VDR-regulated NRF2–GPX4 and NRF2–HO-1 pathways while inducing it by upregulating ATF3 to reduce SLC7A11 expression; VKH2 suppresses lipid peroxidation in an FSP1-dependent manner.

Microbial metabolites directly modulate lipid peroxidation through multiple mechanisms, forming a complex regulatory network that interfaces with the classical ferroptosis pathways (**Figure 4**).

Bile acids exert dual and context-dependent regulation through nuclear receptor signaling. Activation of the FXR upregulates key antioxidant genes such as *GPX4* and *FSP1 (*67), thereby strengthening both the GPX4-GSH and FSP1-CoQ10 defense systems. Simultaneously, FXR induces *SCD1* and *ACSL3*, promoting the synthesis of anti-ferroptotic MUFA-PLs (68), which provide crucial metabolic support for membrane protection. However, under specific conditions, DCA can sensitize cells to ferroptosis by promoting iron accumulation (83), highlighting its dual and context-dependent role. Activation of the vitamin D receptor (VDR) also confers anti-ferroptotic effects via the NRF2/GPX4 and NRF2/HO-1 signaling axis (69, 70), further broadening the regulatory scope of nuclear receptor-mediated control over classical ferroptosis.

SCFAs participate in a multi-layered regulatory network that can either promote or inhibit ferroptosis. Butyrate can downregulate *GPX4* and *SLC7A11* via the FFAR2–mTORC1 axis, thereby promoting ferroptosis (85, 86), constituting a direct intervention on the GPX4-GSH system. Other studies indicate that in lung cancer and osteosarcoma cells, butyrate enhances erastin-induced ferroptosis by regulating the ATF3–SLC7A11 pathway (87, 88). Additionally, butyrate can activate NRF2 through the AMPK–ULK1–p62 signaling pathway, subsequently alleviating ferroptosis in acute liver injury (60). Propionate promotes ACSL4-regulated ferroptosis (70), altering lipid substrate composition to increase cellular sensitivity to ferroptosis. Lactate isomers achieve bidirectional regulation via different pathways: L-lactate inhibits ferroptosis by suppressing AMPK and upregulating SREBP1 and its downstream target gene *SCD1*; whereas D-lactate promotes ferroptosis by downregulating the anti-ferroptosis gene *SLC7A11* and reducing GSH synthesis (65, 89). The two isomers achieve fine-tuning of the ferroptosis threshold through distinct signaling pathways.

Tryptophan-derived metabolites contribute additional layers of protection. Metabolites from the kynurenine pathway act as RTAs while also activating the NRF2 pathway (50, 62), providing both direct antioxidant defense and indirect enhancement of the GPX4-GSH system. 5-HT directly reduces peroxidized phospholipids (62, 96), functioning independently of known classical antioxidant mechanisms. Moreover, IDA reinforces the FSP1-CoQ10 pathway via the AHR-ALDH1A3 signaling axis (63), exemplifying a novel mode through which microbial metabolites modulate ferroptosis defenses.

Vitamin metabolites constitute a crucial antioxidant defense system: Vitamin E, converted to α-tocopherol, can directly scavenge lipid peroxides (75). This action is independent of the GPX4-GSH system, providing an independent antioxidant safeguard. Vitamin D (VD), through VDR, regulates the NRF2–GPX4 and NRF2–HO-1 signaling pathways (69, 74), thereby inhibiting ferroptosis; VD can also induce ferroptosis by upregulating ATF3 transcription to reduce SLC7A11 expression (91). The reduced form of VKH2 inhibits lipid peroxidation in an FSP1-dependent manner (103), forming a regulatory branch parallel and independent to the classical FSP1-CoQ10 system.

#### 3.3.3 Regulation via enzyme systems and pathogen interactions

Microorganisms regulate lipid peroxidation through enzymatic systems and direct host-pathogen interactions, forming complex intersections with the classical ferroptosis pathways. LOXs are core executors of lipid peroxidation, and microbial modulation of LOX activity significantly influences ferroptosis susceptibility. For instance, gut dysbiosis can promote neuroinflammation through activation of 5-LOX, whereas beneficial microbes such as *Acetobacter*, yeast, and *Lactobacillus* exert protective effects by inhibiting 15-LOX activity (54). In contrast, pathogenic bacteria like *Pseudomonas aeruginosa* exploit host 15-LOX to induce ferroptosis directly (78), a mechanism independent of, yet potentially synergistic with, GPX4 dysfunction. Similarly, *Mycobacterium bovis* BCG infection promotes ferroptosis by downregulating *GPX4* and *FSP1* (79), directly impairing two major antioxidant defense systems. Conversely, engineered probiotics such as *Lactococcus lactis* MG1363-pMG36e-GLP-1 have been shown to inhibit ferroptosis via activation of the Keap1/NRF2/GPX4 signaling pathway (55), demonstrating the therapeutic potential of beneficial microbes in reinforcing host ferroptosis resistance.

Collectively, these findings indicate that microbial metabolites and interactions do not simply replicate the actions of classical ferroptosis pathways. Instead, they form a highly integrated defense network that interconnects with canonical systems through independent regulatory modules, metabolic support, and compensatory mechanisms. This multi-layered regulation provides new insight into the physiological and pathological roles of ferroptosis and identifies promising microbial and metabolic targets for therapeutic intervention in ferroptosis-related diseases.

## 4 Ferroptosis and digestive tumors

Ferroptosis plays crucial roles in the proliferation, invasion, and metastasis of gastrointestinal tumors, as well as in the resistance of these tumors to radiotherapy and chemotherapy (**Table 2**).

**Table 2:** Role of ferroptosis in digestive tract tumor.

Type of cancer	Regulating factor	Regulatory mechanism	References
EC	DNAJB6	Exerts a dual tumor-suppressive role by stabilizing GPX4 and activating the AKT1 pathway. Low expression is associated with lymph node metastasis.	(104)
	NRF2	Central role in antioxidant defense. Negative regulation enhances therapy sensitivity; high expression predicts poor prognosis	(105, 106)
	SLC7A11	Key transporter for cystine uptake. High expression correlates with poor prognosis. Inhibitors include sulfasalazine and cisplatin.	(106–108)
	circBCAR3	Promotes tumor proliferation by sponging miR-27a-3p.	(109)
	TMEM161B-AS1	Affects ferroptosis via the miR-27a-3p/GCH1 axis.	(110)
	ARHGEF26-AS1/ADAM23	Depleting protein levels of GPX4, SLC3A2, and SLC7A11.	(111)
	circPVT1	Regulates GPX4 and SLC7A11 via the miR-30a-5p/FZD3 axis.	(112)
GC	CDO1	Modulate core ferroptosis pathways involving ALOX15, SLC7A11, GPX4, and the NRF2/xCT axis	(113–117)
	USP7		
	LncRNA LASTR		
	miR-522		
	Helicobacter pylori (CagA)		
	HIF-1α	Increasing SLC7A11 stability.	(118)
	Artesunate	Elevates intracellular iron to potentiate ferroptosis.	(119)
	Statins	Inhibit selenoprotein synthesis to potentiate ferroptosis.	(7)
HCC	p62/Keap1/NRF2 axis	A critical antioxidant stress pathway that suppresses ferroptosis.	(120, 121)
	Hippo/YAP pathway	Inactivation leads to YAP nucleus translocation, regulating gene expression to inhibit ferroptosis.	(122, 123)
	Wnt/β-catenin axis	Aberrant activation mitigates lipid peroxidation and enhances ferroptosis resistance.	(124, 125)
	Donafinil/GSK-J4	Increases intracellular iron (Fe^2+^) levels.	(126)
	Sorafenib	Induces ferroptosis. Overexpression of S1R confers resistance by modulating the NRF2 pathway and system Xc^−^.	(127, 128)
	Haloperidol	Inhibits S1R, thereby enhancing erastin- and sorafenib-induced ferroptosis by increasing Fe^2+^, depleting GSH, and promoting lipid peroxidation.	(129)
	GSTZ1	Enhances sorafenib-induced ferroptosis by inhibiting the NRF2/GPX4 axis.	(130)
	HSPA8	Up-regulating the expression of SLC7A11/GPX4 and decreasing Erastin-mediated accumulation of ROS and Fe^2+.^	(131)
	RRM2	Stimulates GSH synthesis via GSS.	(132, 133)
	ACSL4	Upregulated in HCC; inhibits erastin-induced ferroptosis via 5-HETE-mediated lipotoxicity.	(134, 135)
	Copper-cyanin	Mediated lipotoxicity.	(136)
	miR-214-3p	Regulating iron homeostasis reduces the expression of ATF4, inhibits GSH synthesis.	(137)
PDA	Cyst(e)inase	Depletes cystine to trigger tumor-selective ferroptosis.	(138)
	GOT1	Inhibition blocks mitochondrial metabolism, promotes a catabolic state, and inhibits GSH synthesis and GPX4 levels.	(139)
	KRAS mutation/Gpx4 knockout	Exhibits a dual role: inducing ferroptosis suppresses tumors, but in specific contexts (e.g., Gpx4 KO), it promotes tumorigenesis via TMEM173/STING1 pathway.	(140)
	Rapamycin + RSL3	Synergistically induce ferroptosis by blocking mTOR and promoting GPX4 degradation.	(141)
	NUPR1 inhibitor (ZZW-115)	Induces ferroptosis associated with mitochondrial dysfunction by downregulating GPX4 and SLC7A11.	(142)
	FBW7	Promotes lipid peroxidation.	(143)
	cTRIP12	Promotes OGT interaction with PERK, enhancing O-GlcNAcylation to stabilize FTH1 and PD-L1, mediating resistance to ferroptosis and immunity.	(144)
	Engineered bacteria	Deplete cysteine to effectively trigger ferroptosis in PDAC cells.	(145)
CRC	P53	Promotes ferroptosis by suppressing SLC7A11 transcription.	(146)
	KIF20A/NUAK1/GPX4 axis	Activation of this axis mediates resistance to oxaliplatin.	(147)
	Fusobacterium nucleatum	Sensitizes cells to oxaliplatin by regulating GPX4.	(148)
	Apatinib	Exerts anti-tumor effect partly by increasing PUFA incorporation into membrane phospholipids.	(149)
	Ferroptosis inducers + Anti-PD-1	Synergistic combination to overcome immunotherapy resistance in MSS-type CRC. Ferroptosis inducers directly kill cells and release DAMPs (e.g., HMGB1) to activate anti-tumor immunity.	(150, 151)
GBC	TFAP2A	Knockdown of *TFAP2A* can inhibit the expression of NRF2.	(152)
	Akt/SIRT3/ACSL4 axis	Affects the ferroptosis process.	(153)
	Isoglycyrrhizin	Activation of the p62-Keap1-NRF2- HMOX1 pathway and down-regulation of GPX4.	(154)
	lithocholic acid	Decreased glutaminase expression.	(155)
	RUNX3	Inhibit SLC7A11.	(156)
OSCC/TSCC/HNSCC	Rhamnosus acid	Suppresses the NRF2/HO-1/xCT pathway.	(157)
	Quisinostat	Elevates ROS via GPX4/p53.	(158)
	Non-thermal plasma	Production of peroxides and mitochondrial superoxide.	(159, 160)
	CircFNDC3B	Sponges miR-520d-5p to suppress SLC7A11.	(161)
	miR-125b-5p/34c-3p	Targeting SLC7A11.	(162–164)
	xCT	Inhibited hypoxia inducible factor (HIF)-1α.	(165)
	PER1	Overexpression inhibits HIF-1α to promote ferroptosis.	(166)
	AEBP1	Silencing activates JNK/p38/ERK signaling to induce cell death.	(166)

### 4.1 Ferroptosis and esophageal cancer

Esophageal carcinoma (EC) remains one of the most prevalent and lethal malignancies worldwide. Treatment of advanced or unresectable EC continues to face the major challenge of resistance to radiotherapy and chemotherapy. In recent years, ferroptosis, an iron-dependent form of regulated cell death characterized by lipid peroxidation, has emerged as a promising mechanism to overcome therapeutic resistance. Evidence suggests that conventional radiotherapy and chemotherapy can induce ferroptosis to a certain degree, and precise modulation of this process may offer an effective strategy to reverse treatment resistance (167).

At the molecular level, ferroptosis is governed by a complex multi-layered regulatory network. GPX4, the core negative regulator of ferroptosis, can be directly inhibited by small molecules such as RSL3 (168), and indirectly modulated by chaperone proteins including HSP27 (169) and DNAJB6. Notably, reduced expression of DNAJB6 is closely associated with lymph node metastasis in EC (104). DNAJB6 exerts a dual tumor-suppressive role by stabilizing GPX4 and activating the signaling AKT1 pathway (170). Concurrently, the transcription factor NRF2 plays a central role in cellular antioxidant defense, and its expression level is a critical determinant of therapeutic response. Downregulation of NRF2 enhances sensitivity to radiotherapy and chemotherapy (105), whereas its overexpression correlates with poor clinical prognosis (106). Natural compounds such as liensinine (171) and polygalacin D (172) have been shown to promote ferroptosis by modulating NRF2 expression. The NRF2-ARF interaction also represents a key mechanism underlying p53-independent ferroptosis (173). SLC7A11, the cystine/glutamate antiporter, functions as another pivotal regulatory hub in ferroptosis. Pharmacological inhibitors of SLC7A11, such as sulfasalazine (107) and cisplatin (108), act through distinct mechanisms to disrupt redox homeostasis. Clinical studies further confirm that elevated SLC7A11 expression is significantly associated with poor prognosis in EC patients (106).

Recent discoveries involving competing endogenous RNA (ceRNA) networks have added a layer of post-transcriptional regulation to ferroptosis in EC. For example, circBCAR3 promotes tumor proliferation by sponging miR-27a-3p (109); TMEM161B-AS1 modulates ferroptosis via the miR-27a-3p/GCH1 axis (110); ARHGEF26-AS1 promotes ferroptosis through the miR-372-3p/ADAM23 pathway (111); and circPVT1 regulates GPX4 and SLC7A11 expression via the miR-30a-5p/FZD3 axis (112). Collectively, these findings reveal a complex post-transcriptional regulatory network that links non-coding RNAs to ferroptosis control, offering novel molecular entry points for the development of targeted therapeutic strategies in esophageal cancer.

In the realm of radio- and chemosensitization, accumulating evidence indicates that ionizing radiation can enhance the susceptibility of EC cells to ferroptosis by upregulating the ferroptosis marker gene *PTGS2* and promoting lipid peroxidation (174). Targeting *SCD1* (175) or epigenetically silencing *METTL3* (176) which affects the m6A modification of *SLC7A11* and *GPX4*, can trigger ferroptosis and improve radiosensitivity. Significant progress has also been achieved in chemotherapy-related ferroptosis research. Multiple conventional anticancer drugs have been shown to promote ferroptosis through diverse mechanisms, including inhibition of system Xc^−^, depletion of intracellular GSH, or modulation of GPX4 activity. For example, *PLK1* knockdown increases lipid peroxidation and enhances sensitivity to paclitaxel and cisplatin (177). Similarly, depletion of HMGA1 (178) or ALDH5A1 (179) disrupts intracellular redox homeostasis, while PARP inhibitors (180) or silencing of *GLRX5* (181) can potentiate cisplatin sensitivity via ferroptosis, particularly in specific genedeficient models.

Therapeutic intervention strategies are becoming increasingly diverse. Small-molecule inhibitors such as erastin and RSL3 play important roles by targeting system Xc^−^ and GPX4, respectively. Inhibition of *SLC7A11* effectively reverses chemoresistance in p53-deficient EC cells (182), while NRF2 inhibitors such as Brusatol (183) enhance radiosensitivity by promoting lipid peroxidation. Natural compounds also display unique advantages. Ferulic acid (184) and oridonin (185) induce ferroptosis by directly inhibiting system Xc^−^ activity, whereas berbamine facilitates GPX4 degradation (186). Alantolactone (187) and licochalcone A (188) indirectly promote ferroptosis by modulating signaling pathways, including p53 and ROS. In addition, realgar (189) and brusatol (190) enhance chemosensitivity by suppressing NRF2 activity, highlighting the therapeutic potential of natural compounds in ferroptosis regulation.

The integration of photodynamic therapy (PDT) and immunotherapy has opened new therapeutic frontiers in EC. Photosensitizers such as talaporfin (191) and 5-aminolevulinic acid (5-ALA) effectively induce ferroptosis by generating ROS and inhibiting the system Xc^−^/GPX4 axis. Moreover, ALA-PDT modulates serum high-mobility group box 1 (HMGB1) levels and macrophage morphology, thereby contributing to enhanced antitumor immunity (192). In immunotherapy, inhibition of NQO1 has been shown to simultaneously induce ferroptosis and increase responsiveness to immune checkpoint blockade (193). Furthermore, advanced nanomaterials offer promising synergistic strategies. For instance, Fe-MOF/CP (194) and 2DG@FS-Nb (195) nanoparticles can deplete intracellular GSH and regulate macrophage polarization states, significantly enhancing anti-tumor immune responses when combined with PD-1 inhibitors.

The advent of nanotechnology has further propelled the development of ferroptosis-based therapies. Iron-based nanozymes effectively induce ferroptosis by catalyzing the Fe³^+^/Fe^2+^ redox cycle and depleting intracellular GSH (196). In addition, various nano-drug delivery systems not only improve drug targeting and bioavailability (197) but also exhibit synergistic therapeutic effects when combined with radiotherapy (198) or chemotherapy (199), achieving precise induction of ferroptosis and amplifying immunogenic cell death in a coordinated manner (200).

In summary, ferroptosis-targeted therapy in EC has progressed from single-target approaches to a new stage of multi-target, synergistic regulation. Close cross-regulation exists among several key molecular pathways. For instance, p53 can induce ferroptosis by inhibiting SLC7A11 (201), while its mutation status influences miR-27a-3p expression (109). Similarly, the Hippo-YAP pathway modulates ferroptosis sensitivity by regulating *TFRC* and *ACSL4* (202) and exhibits reciprocal interactions with *GPX4* and *SLC7A11*. These findings suggest that simultaneously targeting multiple regulatory nodes may yield superior therapeutic outcomes. With the deepening understanding of ferroptosis mechanisms and their integration with conventional modalities such as radiotherapy, chemotherapy, and immunotherapy, ferroptosis-targeted strategies are poised to offer new avenues for improving the prognosis of EC patients.

### 4.2 Ferroptosis and gastric cancer

Ferroptosis, an iron-dependent, lipid peroxidation-driven form of regulated cell death, has emerged as a critical mechanism in gastric cancer (GC), profoundly influencing tumorigenesis, therapeutic responsiveness, and tumor microenvironment (TME) dynamics. The therapeutic induction of ferroptosis holds dual strategic value. First, it directly suppresses tumor proliferation and metastasis. Targeting key negative regulators such as *GPX4*, or applying natural ferroptosis inducers like DHPO, can effectively trigger ferroptotic cell death and synergize with emerging modalities such as CAR-T therapy (113, 203). Second, ferroptosis represents a promising approach to overcoming chemotherapy resistance. Classical inducers such as erastin and RSL3 sensitize GC cells to standard chemotherapeutic agents, including cisplatin (204, 205). Moreover, ferroptosis exerts potent immunomodulatory effects by remodeling the immunosuppressive TME, characterized by elevated ROS levels and reduced expression of immune checkpoints such as PD-L1 (206, 207). The combination of erastin with PD-1 inhibitors demonstrates synergistic tumor clearance, highlighting the dual mechanism of ferroptosis inducers: direct cytotoxicity against cancer cells and enhancement of antitumor immunity.

The execution of ferroptosis in GC is governed by a highly intricate, multi-layered regulatory network. At the molecular level, a diverse array of factors, including non-coding RNAs (e.g., miR-522, LncRNA LASTR), regulatory proteins (e.g., USP7, ATF3, CDO1), and *Helicobacter pylori* infection (through virulence factors such as CagA), precisely modulate core ferroptosis pathways involving ALOX15, SLC7A11, GPX4, and the NRF2/xCT axis (113–117). Within the TME, cellular interactions further shape ferroptotic susceptibility. The elevated iron demand of cancer cells sustains their rapid proliferation, whereas immune infiltrates exert contrasting effects: CD8^+^ T cells can promote ferroptosis by secreting IFN-γ, which downregulates SLC3A2/SLC7A11 (208), while M2-polarized macrophages often suppress ferroptosis and facilitate tumor progression. Moreover, ferroptosis-associated danger signals such as HMGB1 can reinforce this pro-tumor state by promoting M2 macrophage polarization, establishing a self-perpetuating feedback loop (209). Pathophysiological conditions, including hypoxia, hypoxia-induced factor 1α (HIF-1α)/PMAN signaling has been shown to inhibit ferroptosis, thereby conferring survival advantages to tumor cells under oxygen-deprived conditions (118).

The successful clinical translation of ferroptosis-based therapies depends on the identification of reliable biomarkers and the development of innovative therapeutic modalities. Biomarkers such as GPX4 expression levels (210) accumulation of the lipid peroxidation product 4-HNE, and the transport activity of ABCC2 have emerged as valuable indicators for patient stratification, prognosis prediction, and therapeutic monitoring (211, 212). Therapeutically, beyond the classical system Xc^−^ inhibitors (e.g., erastin) and GPX4 inhibitors (e.g., RSL3), multiple agents have been explored to enhance ferroptosis through diverse mechanisms: artesunate increases intracellular iron levels; statins inhibit seleno protein synthesis; and microenvironment-responsive nanocarriers enable targeted delivery of ferroptosis inducers (7, 119, 213, 214). In addition, emerging metabolic targets such as MAT2A exhibit synergistic effects when combined with established ferroptosis inducers, particularly in tumors exhibiting methionine dependency (215).

In summary, targeting ferroptosis represents a promising and multifaceted therapeutic strategy in GC. Nonetheless, several obstacles hinder its clinical implementation, including tumor plasticity that promotes adaptive resistance, limitations in the precision and efficiency of drug delivery systems, and the ferroptosis-suppressive nature of the TME. Future breakthroughs will require a deeper mechanistic understanding of ferroptosis regulation within the TME, alongside the integration of nanotechnology, immunotherapy, and clinical oncology. Such interdisciplinary approaches hold the potential to transform ferroptosis from a mechanistic concept into a clinically controllable and precise anticancer strategy.

### 4.3 Ferroptosis and liver cancer

In hepatocellular carcinoma (HCC), ferroptosis, an iron-dependent form of regulated cell death, has emerged as a significant focus in cancer research. As the central organ responsible for iron metabolism, the liver is particularly vulnerable to disturbances in iron homeostasis, with iron overload acting as a direct trigger of ferroptosis (216). Although the induction of ferroptosis can effectively suppress HCC progression, tumor cells frequently develop adaptive resistance by activating key signaling pathways or altering the expression of ferroptosis-related genes, thereby making ferroptosis both a therapeutic challenge and an attractive target (217).

The core regulatory mechanism of ferroptosis revolves around the cellular antioxidant defense network, with the GPX4-dependent pathway playing a fundamental role (218). The proper function of this system relies on adequate intracellular GSH levels and the enzymatic activity of GPX4. Specifically, the system Xc^−^ transporter, particularly the SLC7A11 subunit, facilitates the uptake of extracellular cystine, a rate-limiting precursor for GSH synthesis (219). GSH serves as a crucial cofactor for GPX4, reducing pro-ferroptotic lipid peroxides into non-toxic lipid alcohols, thereby preventing ferroptotic cell death (218). Consequently, any factor that disrupts system Xc^−^ activity (220), depletes GSH, or inhibits GPX4 function (221) promotes the accumulation of lipid peroxides and ultimately triggers ferroptosis.

Beyond the canonical GPX4 axis, several signaling pathways intricately fine-tune ferroptosis regulation in HCC. The p62/Keap1/NRF2 pathway constitutes a major antioxidant defense mechanism: p62 competitively binds Keap1, leading to NRF2 stabilization and activation. Activated NRF2 upregulates a series of antioxidant and iron metabolism-related genes, collectively suppressing ferroptosis (120, 121). Similarly, inactivation of the Hippo pathway allows its downstream effector YAP to translocate into the nucleus, where it modulates gene expression to inhibit ferroptosis and promote tumor growth (122, 123). Moreover, aberrant activation of the Wnt/β-catenin signaling cascade synergistically enhances ferroptosis resistance in HCC cells, in part by attenuating lipid peroxidation (124, 125). Collectively, these interconnected signaling networks form a complex defense system that enables HCC cells to evade ferroptotic death.

Ferroptosis regulators can be broadly classified into inducers and inhibitors based on their functional roles. Ferroptosis inducers are further categorized by their mechanisms of action into system Xc^−^ inhibitors, GPX4 inhibitors, and GSH-depleting agents (222). Notably, some compounds, such as FINO2, do not directly inhibit GPX4 or system Xc^−^ but instead inactivate GPX4 enzymatic activity indirectly through iron oxidation (223). Conversely, β-mercaptoethanol can bypass system Xc^−^ inhibition by directly facilitating cystine uptake into cells (224, 225). Ferroptosis inhibitors, on the other hand, including endogenous antioxidants, synthetic RTAs, members of the lysyl oxidase family, and iron chelators, can effectively prevent or mitigate ferroptotic cell death. In recent years, non-coding RNAs, including lncRNAs, miRNAs, and circRNAs, have emerged as novel and versatile regulators of ferroptosis. By modulating genes and proteins involved in iron metabolism, lipid metabolism, and antioxidant defense, non-coding RNAs play crucial roles in HCC development, therapeutic resistance, and treatment response (226, 227).

Growing evidence highlights the therapeutic potential of targeting ferroptosis in HCC. For example, the combination of donafenib and GSK-J4 induces ferroptosis by upregulating heme oxygenase-1 (HMOX1), thereby elevating intracellular Fe^2+^ concentrations (126). Conversely, in sorafenib-treated HCC cells, overexpression of the sigma-1 receptor (S1R) reduces oxidative stress via modulation of the NRF2 pathway and system Xc^−^, conferring ferroptosis resistance (127, 128). Conversely, haloperidol-mediated inhibition of S1R enhances erastin- and sorafenib-induced ferroptosis by increasing Fe^2+^ accumulation, depleting GSH, and promoting lipid peroxidation (129). Similarly, the recombinant protein GSTZ1 enhances sorafenib-induced ferroptosis by suppressing the NRF2/GPX4 axis (130). During HBV-associated hepatocarcinogenesis, HSPA8 inhibits ferroptosis by upregulating *SLC7A11* and *GPX4*, thereby reducing erastin-induced ROS and Fe^2+^ accumulation. Inhibition of HSPA8 not only suppresses tumor growth but also sensitizes HBV-positive HCC cells to ferroptosis, highlighting its therapeutic relevance (131). Likewise, the ribonucleotide reductase regulatory subunit M2 (RRM2), frequently overexpressed in HCC tissues, promotes GSH biosynthesis via glutathione synthetase (GSS), thus protecting cells from ferroptosis and contributing to sorafenib resistance (132, 133). Additionally, long-chain ACSL4 is upregulated in HCC and inhibits erastin-induced ferroptosis through 5-hydroxyeicosatetraenoic acid (5-HETE)-mediated lipotoxicity (134, 135). In contrast, inhibition of copper cyanide, a ferroptosis suppressor, enhances Fe^2+^ and ROS accumulation, thereby promoting erastin- and RSL3-induced ferroptosis (136). Non-coding RNAs also play key regulatory roles in HCC ferroptosis. For instance, miR-214-3p promotes ferroptosis by downregulating *ATF4* and inhibiting GSH synthesis (137). Such findings highlight non-coding RNAs as promising molecular targets for modulating ferroptotic sensitivity in HCC.

In summary, ferroptosis serves as a central mechanism in the pathogenesis, progression, and treatment responsiveness of HCC. The regulation of this process involves a highly interconnected network centered on the system Xc^−^/GSH/GPX4 axis, along with multiple signaling pathways, including p62/Keap1/NRF2, Hippo/YAP, and Wnt/β-catenin. Therapeutic strategies that target these pathways, particularly in combination with ncRNA-based interventions or small-molecule modulators, represent a powerful approach for precision treatment of HCC, offering broad prospects for translational research and clinical application.

### 4.4 Ferroptosis and pancreatic cancer

Pancreatic ductal adenocarcinoma (PDAC), a highly aggressive malignancy, develops through a well-characterized progression from normal ductal epithelium to precancerous lesions and ultimately to invasive carcinoma. Although this multistep process is influenced by diverse genetic and environmental factors, the persistent activation of oncogenic KRAS within the inflammatory TME is recognized as the core mechanism (228). Intriguingly, ferroptosis, an iron-dependent, regulated form of cell death characterized by the accumulation of lipid peroxides, was originally identified as a Ras mutation-dependent process upon its discovery (229). This origin-related association suggests the particular therapeutic potential of targeting ferroptosis in KRAS-mutant pancreatic cancer. However, accumulating evidence has revealed a paradoxical duality in the role of ferroptosis within PDAC: under certain conditions, it acts as a potent tumor-suppressive mechanism, whereas in others, it paradoxically promotes tumor initiation and progression.

This dual nature has been clearly demonstrated in multiple genetically engineered mouse models (GEMMs). In the KPFSR model, tamoxifen-induced systemic deletion of *Slc7a11* triggered tumor-selective ferroptosis and significantly inhibited tumor growth. Similarly, cystine depletion using cyst(e)inase reproduced significant tumor suppression in the KPC model (138). Mechanistically, beyond directly disrupting cystine uptake, targeting downstream metabolic nodes can also effectively induce ferroptosis. For instance, inhibition of cytosolic aspartate aminotransferase disrupts cellular metabolic balance and enhances autophagic flux, increases the labile iron pool, and ultimately promotes ferroptotic cell death (139). Paradoxically, in pancreas-specific *Gpx4* knockout KC models or under high-iron diet conditions, ferroptosis instead facilitated tumorigenesis (140). Mechanistic investigations revealed that ferroptosis-induced oxidative DNA damage results in the release of 8-hydroxyguanosine (8-OHG), which activates the TMEM173/STING1-dependent DNA sensing pathway, promoting macrophage recruitment and activation during the early stages of tumor formation. Another study demonstrated that autophagy-dependent ferroptosis enables tumor cells to release mutant KRASG12D protein via exosomes, driving macrophage polarization toward the pro-tumorigenic M2 phenotype (140). This seemingly contradictory behavior of ferroptosis in PDAC likely depends on contextual factors, including differences in genetic background (such as TP53 mutation status) and the pleiotropic functions of key regulators like GPX4 and SLC7A11, which participate in both ferroptosis-dependent and ferroptosis-independent cellular pathways.

The influence of ferroptosis in pancreatic cancer extends well beyond tumor cell death; it profoundly reshapes the tumor immune microenvironment through the release of diverse signaling molecules. On one hand, ferroptosis can promote M2-type tumor-associated macrophage (TAM) polarization via the release of KRAS^G12D^, thereby promoting an immunosuppressive milieu (140). On the other hand, ferroptosis can also activate anti-tumor immune responses by stimulating the production of pro-inflammatory cytokines in an NF-κB-dependent manner. This occurs through the release of decorin, which binds to the AGER receptor on macrophages, driving immune activation (230). Similarly, HMGB1, released during ferroptosis, functions as a damage-associated molecular pattern (DAMP) molecule recognized by antigen-presenting cells, triggering innate and adaptive immune responses (209). Collectively, these findings indicate that the net immunological outcome of ferroptosis in pancreatic cancer depends on the immune microenvironment depends on multiple contextual factors, including the specific signaling molecules released, their spatiotemporal dynamics, and the phenotypes of the immune cells that receive these signals. Although bioinformatics analyses have preliminarily constructed association networks linking ferroptosis regulators to patterns of immune cell infiltration (231), these correlations remain predictive and require rigorous experimental validation.

Given the broad resistance of pancreatic cancer to conventional chemotherapy, therapeutically inducing ferroptosis has emerged as a promising approach to overcome drug resistance. Targeting key regulatory nodes has shown encouraging results: the combination of rapamycin and RSL3 efficiently induces ferroptosis by synergistically inhibiting mTOR signaling and promoting GPX4 degradation (141). Drug repositioning also holds potential: zalcitabine, for instance, induces mitochondrial DNA stress and activates the STING1 pathway, leading to autophagy-dependent ferroptosis (232). Similarly, the NUPR1 inhibitor ZZW-115 triggers ferroptosis associated with mitochondrial dysfunction by downregulating *GPX4* and *SLC7A11* expression (142). Among natural products, a triple combination of piperlongumine, cortinin A, and sulfasalazine demonstrates synergistic efficacy in inducing ferroptosis (233). Moreover, artesunate and its active metabolite dihydroartemisinin exhibit selective cytotoxicity against RAS-mutant pancreatic cancer cells, primarily through mechanisms involving iron metabolism dysregulation (234). In terms of combination therapies, ferroptosis inducers such as erastin and RSL3 can enhance the cytotoxicity of gemcitabine (143), while the co-administration of dihydroartemisinin and cisplatin exerts synergistic anti-tumor effects by simultaneously inducing ferroptosis and exacerbating DNA damage (235).

Chemotherapy resistance remains a major obstacle in the clinical management of pancreatic cancer, and the ferroptosis pathway plays a complex and context-dependent role in this process. Tumor cells can evade ferroptotic death by activating adaptive stress responses. For instance, the gemcitabine-induced ATF4-HSPA5 axis stabilizes GPX4, thereby suppressing ferroptosis and promoting drug resistance (236). To overcome these resistance mechanisms, several sensitization strategies have been developed. The tumor suppressor FBW7 inhibits *SCD1* expression by promoting NR4A1 degradation, which alters membrane lipid composition and enhances susceptibility to lipid peroxidation (143). TRIM21 counteracts ferroptosis by ubiquitinating and degrading the arachidonic acid metabolism enzyme EPHX1, a process that can be pharmacologically targeted by bezafibrate (237). In KRAS-mutant tumors, the KRAS/ERK1 axis promotes ALOX15B degradation via ABHD17C, while methylprotodioscin disrupts this interaction to restore ALOX15B’s pro-ferroptotic activity (238). A particularly noteworthy finding involves the circular RNA cTRIP12, which facilitates the interaction between OGT and PERK, thereby enhancing O-GlcNAcylation and stabilizing ferritin heavy chain and PD-L1. This dual stabilization mediates resistance to both ferroptosis and immune checkpoint therapy, making cTRIP12 a promising target for combination treatment (144). Additional resistance mechanisms have also been elucidated. KRAS mutations can upregulate TMOD3, promoting F-actin polymerization, which in turn enhances autophagosome–lysosome fusion and accelerates ACSL4 degradation. This process inhibits ferroptosis and contributes to resistance against PD-1 antibodies (239). In parallel, targeting cysteine metabolism, a critical component of ferroptosis defense, has shown promise. Engineered bacteria designed to deplete cysteine effectively induce ferroptosis in PDAC cells by precisely disrupting cysteine utilization (145).

In summary, ferroptosis exerts a context-dependent dual role in pancreatic cancer: it can act as a potent tumor-suppressive mechanism, directly eliminating malignant cells, yet under certain conditions, it can also release pro-tumorigenic signaling molecules that reshape the tumor microenvironment. Preclinical studies have demonstrated the potential of ferroptosis-targeted therapies to overcome both apoptosis and immune resistance. Future research should focus on identifying the molecular switches that dictate the pro- or anti-tumor fate of ferroptosis, elucidating its immunogenic mechanisms, and developing low-toxicity, clinically translatable ferroptosis inducers for optimized combination therapies.

### 4.5 Ferroptosis and colorectal cancer

Ferroptosis, an iron-dependent, lipid peroxidation-driven form of regulated cell death, plays a central role in the initiation, progression, and therapeutic resistance of colorectal cancer (CRC). Its core regulatory mechanism revolves around the system Xc–GPX4 antioxidant axis, the disruption of which directly leads to the accumulation of lipid peroxides and subsequent cell death. In CRC, a complex molecular network finely regulates this process. For instance, p53 promotes ferroptosis by suppressing SLC7A11 transcription (146), whereas activation of the NRF2 signaling pathway enables cancer cells to evade ferroptosis through SLC7A11 upregulation (240). Furthermore, the KIF20A/NUAK1/GPX4 axis has been identified as a critical mechanism conferring resistance to oxaliplatin-based chemotherapy (147). A profound bidirectional relationship exists between ferroptosis and therapeutic resistance: while certain targeted drugs may inadvertently inhibit ferroptosis and promote drug resistance, the deliberate induction of ferroptosis represents a promising approach to overcoming such resistance. Beyond its direct cytotoxic effects on tumor cells, ferroptosis also influences the TME by releasing diverse signaling molecules that remodel immune responses and correlate closely with patient prognosis.

Within the unique setting of the colorectum, the gut microbiota serves as a crucial “external regulator,” adding a layer of complexity to ferroptosis modulation. Microbially derived metabolites, including vitamins, bile acids, SCFAs, and tryptophan metabolites, exert highly context-dependent, bidirectional effects on ferroptosis. Most vitamins and certain bile acids inhibit ferroptosis by upregulating GPX4 or activating antioxidant pathways such as NRF2, thereby potentially enhancing tumor survival. In contrast, SCFAs (such as butyrate) tend to promote ferroptosis and suppress tumor growth by downregulating *SLC7A11* and *GPX4* expression. Interestingly, some metabolites, such as VB2 and specific bile acids, display dual functions, either promoting or suppressing ferroptosis depending on the microenvironmental context (11). Collectively, this intricate microbiota-mediated regulation of ferroptosis enriches our understanding of host–microbe interactions in colorectal cancer and highlights promising opportunities to modulate ferroptosis through dietary, probiotic, or microbial-targeted interventions.

Building on these mechanistic insights, targeting the key metabolic nodes of ferroptosis, namely, iron, amino acid, and lipid metabolism, has emerged as a highly promising therapeutic strategy. At the iron metabolism level, modulating the activity of proteins such as the transferrin receptor or ferritin to expand the intracellular LIP effectively induces ferroptosis, a mechanism exploited by agents like vitamin C (241). Within amino acid metabolism, the system Xc–GPX4 axis represents the central therapeutic target. Compounds such as erastin, which inhibits SLC7A11, or RSL3, which directly inactivates GPX4, can robustly trigger ferroptotic cell death. Notably, the gut bacterium Fusobacterium nucleatum has been shown to enhance oxaliplatin sensitivity by modulating *GPX4* expression (148). Regarding lipid metabolism, the promotion of PUFA incorporation into membrane phospholipids, a process catalyzed by ACSL4, provides the essential biochemical “fuel” for ferroptosis (242). Indeed, drugs such as apatinib exert part of their anti-tumor efficacy through this pathway (149).

Perhaps even more compelling is the combination of ferroptosis inducers with immune checkpoint inhibitors, which has opened a new frontier for overcoming immunotherapy resistance in CRC, particularly in microsatellite stable (MSS) patients (150). This combined strategy operates through multiple synergistic mechanisms: ferroptosis inducers (e.g., RSL3, sorafenib) directly promote tumor cell death (243, 244), while the release of DAMPs, such as HMGB1, from ferroptotic cells activates dendritic cells and recruits CD8^+^ T lymphocytes, transforming an immunosuppressive “cold” tumor microenvironment into an immunostimulatory “hot” one (151). Cutting-edge research is now focused on identifying natural ferroptosis-inducing compounds from traditional medicines, such as artemisinin and ginsenoside Rh3, that act through multiple regulatory pathways (245), as well as developing intelligent nanodelivery systems capable of tumor-targeted co-delivery of ferroptosis inducers and immunotherapeutic agents (246). These advances aim to precisely modulate tumor metabolism while simultaneously triggering robust anti-tumor immunity, heralding a new era of integrated metabolic intervention and immunotherapy in CRC treatment.

### 4.6 Ferroptosis in other digestive cancers

Compared with the major digestive system malignancies discussed above, research on ferroptosis in gallbladder cancer (GBC) and oral cavity cancer, particularly oral squamous cell carcinoma (OSCC), remains in its infancy. Current evidence is largely derived from preliminary *in vitro* studies, and the field is still primarily descriptive, focusing on the identification of potential ferroptosis regulators rather than the construction of comprehensive mechanistic networks or translational frameworks.

In GBC, several molecular factors have been implicated in ferroptosis regulation. Induction of ferroptosis appears to be promoted by *TFAP2A* knockdown (which suppresses NRF2) (152), activation of the Akt/SIRT3/ACSL4 axis (153), and treatment with compounds such as isoglycyrrhizin, which acts through the p62-Keap1-NRF2-HMOX1 pathway and GPX4 downregulation (154), or lithocholic acid, which inhibits glutaminase and depletes GSH (155). Conversely, RUNX3 may inhibit ferroptosis via a p53-dependent mechanism that activates GADD45A and subsequently suppresses *SLC7A11* expression (156).

In OSCC, several regulators have also been identified that modulate sensitivity to treatments such as cisplatin and PDT. Ferroptosis inducers include rhamnazin (which inhibits the NRF2/HO-1/xCT pathway) (157), Quisinostat (which elevates ROS via the GPX4/p53 axis) (158), and non-thermal plasma (159, 160). Resistance mechanisms, on the other hand, involve molecules such as circFNDC3B and specific miRNAs (e.g., miR-125b-5p, miR-34c-3p) that target and suppress SLC7A11/xCT (161–164), as well as activation of the IL-6/JAK2/STAT3 signaling pathway (165). Furthermore, PER1 overexpression promotes ferroptosis by inhibiting HIF-1α, while AEBP1 silencing activates JNK/p38/ERK signaling to induce cell death (166). Collectively, these findings provide preliminary evidence that ferroptosis plays a functional role in GBC and OSCC; however, further *in vivo* validation and mechanistic dissection are essential to clarify its biological significance and assess the therapeutic potential of ferroptosis-targeted interventions in these cancers.

## 5 Ferroptosis in tumor therapy

The high uptake of iron by tumors and the bidirectional effect of iron metabolism on tumor microenvironment provide more means for clinical anti-tumor cells. Ferroptosis has become a key new target for tumor therapy.

### 5.1 Chemotherapy and ferroptosis

Chemotherapy remains a primary treatment for malignant tumors, but its efficacy is often limited by drug resistance. Recent studies have indicated that ferroptosis is closely associated with chemotherapy resistance. Combining ferroptosis inducers with chemotherapeutic agents may help overcome clinical challenges and improve therapeutic outcomes. In gastric cancer, STAT3 acts as a negative regulator of ferroptosis. The STAT3 inhibitor W1131 has demonstrated anti-tumor effects by inducing ferroptosis and may synergize with chemotherapy to further suppress tumor growth (247). Similarly, kaurane-type diterpenoids promote both ferroptosis and apoptosis by inhibiting peroxidase activity of Prdx I/II, and exhibit synergistic anti-tumor effects when combined with cisplatin (248). Sorafenib, a first-line treatment for advanced hepatocellular carcinoma, often faces resistance issues. Metallothionein IG (MTIG), involved in oxidative stress response, inhibits SRF-induced ferroptosis. NRF2 activation is crucial for MTIG expression, and targeting this pathway may help attenuate SRF resistance (249, 250). However, chemotherapy-induced ferroptosis may also damage normal tissues (251). Thus, balancing cancer cell elimination with protection of healthy cells is essential in treatment design. **Table 3** summarizes ferroptosis-targeting agents currently used clinically or with strong translational potential.

**Table 3:** Application of ferroptosis-related drugs in tumor therapy.

Drug	Target molecules	Regulatory mechanism	References
Sulfasalazine	SLC7A11	Inhibits system Xc^−^, depletes GSH	(107)
Sorafenib	SLC7A11	Promotes ferroptosis by inhibiting system Xc^−^	(243)
Erastin	SLC7A11, GPX4	Inhibits system Xc^−^, leading to cysteine starvation, GSH depletion, and GPX4 inactivation	(252)
Paclitaxel	SLC7A11, P53	Inhibits lipid peroxidation; Sensitizes cells to ferroptosis when combined with *PLK1* knockdown	(177)
Brusatol	NRF2	Inhibits NRF2, promotes lipid peroxidation, and enhances radiosensitivity	(183) (190)
Fin56	GPX4	Degrades GPX4; activates squalene synthase	(8)
(1S, 3R)-RSL3	GPX4	Directly inhibits GPX4	(2)
Statins	GPX4, HMGCR, CoQ10	Inhibits HMGCR, decreases GPX4 level, blocks biosynthesis of CoQ10 via the mevalonate pathway, inhibits the expression of PD-L1	(119)
Gemcitabine	GPX4	Inhibits lipid peroxidation; but can induce an adaptive ATF4-HSPA5 response that stabilizes GPX4 and confers resistance	(236)
Dihydroartemisinin (DHA)	GPX4, Ferritin	Inhibits GPX4 synthesis and induces iron metabolism; synergizes with cisplatin	(235)
Artemisinin derivatives (e.g., Artesunate)	Ferritin, Iron Metabolism	Enhances lysosomal degradation of ferritin; increases intracellular iron levels; specifically cytotoxic to Ras-mutant cells	(234)
Deferoxamine	Iron	Iron chelator, suppresses ferroptosis	(3)
L-buthionine-sulfoximine (BSO)	GSH	Inhibits γ-glutamylcysteine synthetase, depleting GSH	(4)
Cisplatin	GSH, SLC7A11	Depletes GSH; can promote ferroptosis via SLC7A11 inhibition	(108)
Brequinar (BRQ)	DHODH	Inhibits mitochondrial DHODH, promotes ferroptosis especially in GPX4-low cancer cells	(38)
Haloperidol	Sigma-1 Receptor (S1R)	Inhibits S1R, enhances erastin- and sorafenib-induced ferroptosis by increasing Fe^2+^, depleting GSH, and promoting lipid peroxidation	(129)
ZZW-115	NUPR1	Inhibits NUPR1, downregulates GPX4 and SLC7A11, induces mitochondrial dysfunction-associated ferroptosis	(142)
Zalcitabine	Mitochondria, STING1	Induces mitochondrial DNA stress, activates the STING1 pathway, leading to autophagy-dependent ferroptosis	(232)
Cyst(e)inase	Cyst(e)ine	Degrades extracellular cysteine and cystine, depleting GSH and inducing ferroptosis	(138)
Ferrostatin-1, Liproxstatin-1	Lipid Peroxides	Potent ferroptosis inhibitors; act as RTAs	(9)
Vitamin E	Lipid Peroxides	Converted to α-tocopherol, directly scavenges lipid peroxides as an RTA	(75)

### 5.2 Immunotherapy and ferroptosis

Immunotherapy achieves anti-tumor effects by activating the immune system and enhancing one’s ability to fight cancer. Ferroptosis not only has direct anti-tumor effects, but also enhances the immunogenicity of cancer cells and the sensitivity of immunotherapy, bringing new opportunities for tumor treatment, especially in addressing the problem of drug resistance to immunotherapy drugs (252). Immunocheckpoint inhibitors (ICIs) mainly promote lipid peroxidation-dependent ferroptosis of tumor cells by activating CD8^+^ T cells, releasing IFN-γ, down-regulating the expression of SLC7A11 and SLC3A2 (208). Clinically, ACSL4 has improved the survival rate of cancer patients treated with ICIs (253). Similarly, in melanoma patients, a decrease in SLC3A2 expression is strongly associated with an increase in the efficacy of ICIs (254). Damage-associated molecules released by ferroptosis cells promote dendritic cell maturation and induce CD8^+^ T cell activation. Activated CD8^+^ T cells inhibit ferroptosis by binding to TYRO3 and promote the development of a pro-tumor microenvironment by decreasing the M1/M2 macrophage ratio, leading to resistance to anti-PD-1/PD-L1 therapy (255). Studies have found that anti-PD-1 drugs and TGFβ inhibitors can synergistically enhance the immune response in TME, resulting in an increase in the content of H_2_O_2_ in M1 macrophages, promoting Fenton reaction, and inducing ferroptosis of tumor cells (256). It is hypothesized that the long-term effects of ferroptosis on tumor immunotherapy depend on the interaction between tumor cells and various immune cell subsets.

### 5.3 Radiotherapy and ferroptosis

Radiotherapy produces ROS and free radicals through ionizing radiation to destroy chemical bonds, leading to DNA damage and exerting anti-tumor effects. Radiotherapy significantly induced the expression of ACSL4, which in turn promoted ferroptosis of tumor cells, increased the sensitivity of tumors to radiotherapy, and effectively inhibited tumor growth (174). ACSL4 deletion or treatment with liproxstatin-1, an inhibitor of ferroptosis, significantly reduced 4-hydroxynonenal (4-HNE) levels, while the level of 4-HNE in esophageal cancer tissues after radiotherapy was significantly associated with better clinical prognosis of cancer patients (257). Radiotherapy can induce the expression of SLC7A11 and GPX4, and promote radioresistance by inhibiting ferroptosis. Inhibition of SLC7A11 or GPX4 with ferroptosis inducers can increase the sensitivity of radioresistant tumor cells and xenografts to radiotherapy (174). Hypoxia microenvironment is an important feature of solid malignant tumors. Radioresistance caused by hypoxia is the main reason for its poor radiotherapy effect (258, 259). In addition, the up-regulation of HIF-1α caused by hypoxia is also an important factor in radioresistance (260, 261). HIF-1α-mediated ferroptosis inhibition is closely related to poor prognosis and treatment tolerance of hypoxic tumors. Therefore, regulating ferroptosis to reduce HIF-1α-induced radioresistance may be an effective strategy (164). Whole lactoferrin can down-regulate the expression of HIF-1α, improve the hypoxic microenvironment of breast cancer, and promote radiation-induced DNA damage, improving the radiotherapy effect of breast cancer (262). In addition, studies have found that IFN-γ released by immunotherapy-activated CD8^+^ T cells and radiotherapy-activated macrophages can independently and synergistically inhibit SLC7A11.This leads to a decrease in cystine uptake, which in turn increases lipid peroxidation and ferroptosis, making tumor cells sensitive to radiotherapy (263). Therefore, in clinical practice, radiotherapy is usually combined with chemotherapy, targeted therapy or immunotherapy to enhance the clearance of tumor cells.

### 5.4 Nanomedicine and ferroptosis

Nanotechnology has achieved remarkable success in cancer treatment. The ferroptosis mediated by Fe_3_O_4_-SAS @ PLT assembled by sulfasalazine (SAS) -loaded magnetic nanoparticles (Fe_3_O_4_) and platelet (PLT) membrane can not only induce tumor-specific immune response, but also effectively repolarize immunosuppressive M2 into anti-tumor M1, regulating the anti-tumor interaction between tumor cells and macrophages (264). In the nanoparticle SRF@Hb-Ce6, which constructed by connecting hemoglobin (Hb) with photosensitizer chlorin e6 (Ce6) and loading sorafenib, Hb utilizes its own oxygen and iron to provide oxygen for PDT while providing iron for ferroptosis. PDT enhances ferroptosis by inducing IFN-γ secretion by immune cells (265). In addition, the nanoparticles SRF @ MPDA-SPIO which constructed based on superparamagnetic iron oxide (SPIO) also have the functions of inducing ferroptosis and PDT (266). Polyethylene glycol-modified ultra-small silicon nanoparticles (C′ dots) for tumor imaging can bind and enrich iron ions, which can be endocytosed into cells and lead to an increase in intracellular iron content, causing iron metabolism disorders in tumor cells (267, 268). Ultrasmall single-crystal Fe nanoparticles (bcc-USINP) were synthesized by one-step high-temperature pyrolysis, providing a simple, safe and efficient tumor-responsive zero-valent iron (Fe) delivery system, which can selectively release a large amount of iron ions in weakly acidic TIME and promote Fenton reaction. Bcc-USINP can cause ferroptosis of tumor cells at lower concentrations (269). Nanomaterials have the advantages of promoting drug dissolution, improving absorption and precise targeting, and show great promise for combining them with ferroptosis in cancer treatment.

## 6 Conclusion

During the development and therapeutic response of digestive tract cancers, ferroptosis—an iron-dependent, programmed form of cell death intricately regulated by the gut microbiota and their metabolites—plays a critical role. In recent years, mechanistic studies of ferroptosis in gastrointestinal tumors have made substantial progress, gradually uncovering the key molecules and signaling pathways involved. However, the interplay between ferroptosis and other regulated cell death pathways, such as autophagy and apoptosis, remains incompletely understood. Moreover, clinical detection and application of ferroptosis-related biomarkers or therapies are still at an early stage, and the development of ferroptosis-targeted anti-cancer drugs remains limited. This persistent gap between basic research and clinical translation continues to impede the full recognition and therapeutic utilization of ferroptosis in GI cancer management.

Based on the evidence presented, we propose a coherent integrative model in which gut microbial metabolites serve as central conductors, fine-tuning the ferroptosis orchestra within the tumor microenvironment. This model posits that the balance between ferroptosis-suppressive and ferroptosis-promoting signals from the gut microbiota can decisively influence cancer cell fate.

The model highlights two primary, opposing forces. On one hand, beneficial microbes and their metabolites primarily act as ferroptosis inhibitors, which can paradoxically support tumor cell survival. Key mechanisms include: SCFAs like butyrate, which enhance mitochondrial function and activate the cytoprotective NRF2 pathway; bile acids that activate nuclear receptors (FXR/VDR) to upregulate a ferroptosis-defense network; tryptophan derivatives that activate the AHR receptor to bolster cellular antioxidant systems; and vitamin metabolites that provide crucial redox regulation - VK forms a defense branch independent of GPX4 through an FSP1-dependent pathway, while vitamin A and E derivatives act as direct radical-trapping antioxidants providing chemical protection; and probiotics that help maintain systemic iron homeostasis and activate protective pathways.

In contrast, gut dysbiosis and pathogenic bacteria can drive ferroptosis, a process that can be harnessed for therapy or contribute to tissue damage. This occurs through several mechanisms: Pathogen-associated molecules like LPS and specific bacteria can upregulate pro-ferroptotic enzymes such as ACSL4 and ALOX15; altered bile acid profiles can promote iron uptake and lipid peroxidation; the depletion of protective metabolites like SCFAs indirectly lifts the brakes on ferroptosis; and notably, VB2 (VB2) demonstrates context-dependent duality - under specific microenvironmental conditions (such as when forming nanocomplexes with iron ions), VB2 can transform from a “protector” into an “attacker,” synergistically promoting iron accumulation and ROS generation, thereby exhibiting pro-ferroptotic activity.

This dual perspective positions the gut microbiota at the core of ferroptosis regulation, offering novel strategies for cancer treatment. Interventions such as probiotics, prebiotics, fecal microbiota transplantation, direct administration of pro-ferroptotic metabolites, or elimination of ferroptosis-suppressive microbes can modulate the balance of ferroptosis—suppressing it in normal tissues to reduce damage, or selectively inducing it in tumors to overcome drug resistance.

While the potential of harnessing the gut microbiome to modulate ferroptosis is a compelling strategy for cancer therapy, it is crucial to acknowledge the significant limitations of the current research landscape. The evidence summarized herein is predominantly derived from *in vitro* cell cultures and animal models. These pre-clinical systems cannot fully replicate the complexity of the human tumor microenvironment or the dynamic ecosystem of the human gut, creating a substantial translational gap before these findings can be applied clinically.

A pivotal, yet often unaddressed, question is the physiological relevance of the microbial metabolites involved. Many studies utilize supraphysiological concentrations to elicit clear ferroptotic responses *in vitro*. It remains unclear whether the local or systemic levels of metabolites like SCFAs, bile acids, or tryptophan derivatives in humans are sufficient to significantly influence ferroptosis pathways in tumors. Furthermore, human data are largely correlative, making it difficult to establish causality between microbial shifts and ferroptosis modulation in cancer patients.

To advance this field, future research must bridge these translational gaps. Priority directions include conducting human studies that integrate microbiome and metabolomic profiling with ferroptosis biomarkers in patient biopsies; determining the pharmacologically and physiologically achievable concentrations of key microbial metabolites, and launching early-phase clinical trials that combine ferroptosis inducers with microbiome-targeted interventions. Addressing these limitations will be essential to building a realistic and mechanistically grounded framework for translating ferroptosis-based strategies into clinical oncology.

Overall, ferroptosis plays a fundamental role in the proliferation, invasion, metastasis, and resistance to radiotherapy and chemotherapy in gastrointestinal cancers, highlighting its immense potential as a therapeutic target. As research continues to unravel the intricate regulatory networks governing ferroptosis and its interplay with the tumor microenvironment, this form of cell death is poised to become a central target for improving clinical outcomes in patients with gastrointestinal malignancies. Harnessing the microbiota-metabolism-ferroptosis axis for metabolic intervention may serve as a crucial bridge between basic discovery and clinical translation, paving the way for next-generation, precision cancer therapies.
